# The long‐term impact and value of curative therapy for HIV: a modelling analysis

**DOI:** 10.1002/jia2.26170

**Published:** 2023-09-25

**Authors:** Gregory F. Guzauskas, Timothy B. Hallett

**Affiliations:** ^1^ The Comparative Health Outcomes, Policy, and Economics Institute Department of Pharmacy University of Washington Seattle Washington USA; ^2^ HCD Economics Daresbury UK; ^3^ MRC Centre for Global Infectious Disease Analysis Imperial College London London UK

**Keywords:** antiretroviral therapy (ART), cost‐utility analysis (CUA), curative therapy (CTx), human immunodeficiency virus (HIV), low‐ and middle‐income countries (LMICs), value‐based price (VBP)

## Abstract

**Introduction:**

Curative therapies (CTx) to achieve durable remission of HIV disease without the need for antiretroviral therapy (ART) are currently being explored. Our objective was to model the long‐term health and cost outcomes of HIV in various countries, the impact of future CTx on those outcomes and the country‐specific value‐based prices (VBPs) of CTx.

**Methods:**

We developed a decision‐analytic model to estimate the future health economic impacts of a hypothetical CTx for HIV in countries with pre‐existing access to ART (CTx+ART), compared to ART alone. We modelled populations in seven low‐and‐middle‐income countries and five high‐income countries, accounting for localized ART and other HIV‐related costs, and calibrating variables for HIV epidemiology and ART uptake to reproduce historical HIV outcomes before projecting future outcomes to year 2100. Health was quantified using disability‐adjusted life‐years (DALYs). Base case, pessimistic and optimistic scenarios were modelled for CTx+ART and ART alone. Based on long‐term outcomes and each country's estimated health opportunity cost, we calculated the country‐specific VBP of CTx.

**Results:**

The introduction of a hypothetical CTx lowered HIV prevalence and prevented future infections over time, which increased life‐years, reduced the number of individuals on ART, reduced AIDS‐related deaths, and ultimately led to fewer DALYs versus ART‐alone. Our base case estimates for the VBP of CTx ranged from $5400 (Kenya) up to $812,300 (United States). Within each country, the VBP was driven to be greater primarily by lower ART coverage, lower HIV incidence and prevalence, and higher CTx cure probability. The VBP estimates were found to be greater in countries where HIV prevalence was higher, ART coverage was lower and the health opportunity cost was greater.

**Conclusions:**

Our results quantify the VBP for future curative CTx that may apply in different countries and under different circumstances. With greater CTx cure probability, durability and scale up, CTx commands a higher VBP, while improvements in ART coverage may mitigate its value. Our framework can be utilized for estimating this cost given a wide range of scenarios related to the attributes of a given CTx as well as various parameters of the HIV epidemic within a given country.

## 1 INTRODUCTION

Over the past three decades, the use of combination antiretroviral therapy (ART) has diminished global HIV mortality and transformed the disease into a chronic and manageable condition in geographies where ART is accessible and patients are adherent [1]. Nonetheless, HIV remains prevalent in vulnerable populations, particularly those in low‐ and middle‐income countries (LMICs) in sub‐Saharan Africa, and may remain so in the absence of an intervention that leads to durable ART‐free suppression of viraemia (“cure”) and that is also accessible and affordable [2].

Assuming that technical, practical and safety hurdles can be overcome, it is anticipated that curative therapy (CTx) for HIV infection may be available within the next 10–20 years [3, 4]. At that point, the looming hurdles to their global uptake are likely to be related to pricing and financing, which will be complicated if CTx consists of a one‐time administration with an upfront payment, similar to current gene therapies [5]. Understanding the economic value of CTx in different contexts will be key to unlocking questions of funding feasibility and negotiating a fair price for each country that wishes to access it [6, 7, 8, 9].

It has been previously argued that health technology interventions are public economic goods, and that the optimal research and design financing system should be based on global differential pricing across countries that accounts for both the ability and the willingness‐to‐pay for improved population health [10]. Danzon and others have further argued that if each country/payer sets a cost‐effectiveness threshold based on the marginal health opportunity costs, and manufacturers subsequently price their products up to or below each threshold (after allowing for costs of delivery), the resulting prices and utilization would be “value‐based” and yield sustainable global incentives for future utilization and innovation [11, 12].

Given the substantial costs of research and development for novel CTx therapies as well as challenges associated with their production, distribution, delivery and administration, there is a need to further investigate what the properties and price for the technology would be, consistent with this perspective. Our first objective was to develop a decision‐analytic model that can be adjusted to specific countries’ HIV epidemiology, ART utilization, demographics and economic conditions to estimate long‐term HIV‐related costs and health outcomes. Our second objective was to use the model to assess the potential HIV epidemic impacts of a future CTx once it becomes available. Our third objective was to estimate the *value‐based price* (VBP) appropriate for each country to pay per CTx administration based on their unique cost‐effectiveness thresholds [13].

## 2 METHODS

### 2.1 Decision model

Decision‐analytic state‐transition modelling is a common health economic evaluation approach that can be applied to different populations and diseases [14, 15]. Modelled individuals or proportions of modelled cohorts reside within “health states” that correspond to specific stages of disease natural history for a given time period. The time spent in each health state can be used in conjunction with state values (e.g. life‐years, health state‐specific disability weights and costs) to estimate life expectancy, disability‐adjusted life‐years (DALYs) and expected costs [15].

**Figure 1:**
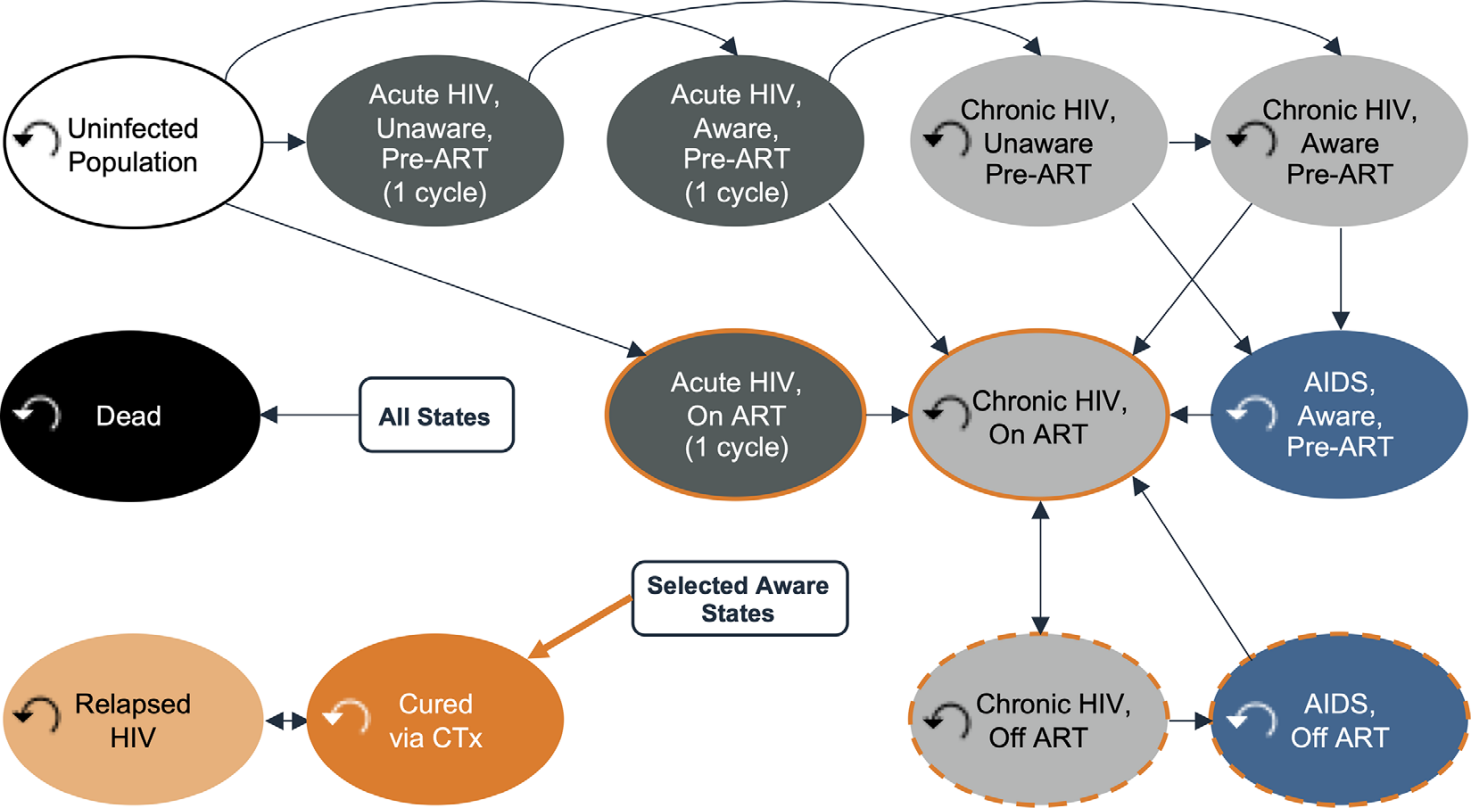
HIV state transition model. Individuals could transition from uninfected to an HIV health state or from one HIV health state to another. Individuals automatically transitioned from early HIV infection to chronic HIV after a single semi‐annual model cycle. In the CTx+ART comparator arm, eligible individuals could receive CTx. As determined, however, by the modelled durability of CTx cure, they could transition to a relapsed HIV health state with costs and disability weights equivalent to chronic HIV until they either received another CTx administration or died. Individuals could transition to death via AIDS‐associated mortality or the background mortality of the modelled country.

We developed an open cohort state‐transition model [16], which (in contrast to closed cohort models) allows for new individuals to enter the analysis in each model cycle, to compare the introduction of a novel CTx for HIV in settings with pre‐existing access to ART (CTx+ART) versus ART alone (Figure 1). The model was developed in Microsoft Excel and is available for download here. All model parameter data were collected from October 2021 to July 2023 and can be found in online Appendix Table A1 as well as citations within the model itself. We made assumptions where data were lacking and have documented these instances as such. We utilized a semi‐annual model cycle duration and a healthcare payer perspective, and we discounted all future HIV‐related cost and health outcomes by a constant 3% per year to reflect their present value [14].

The model incorporates 2010–2021 UNAIDS HIV epidemiological estimates [17, 18] for each modelled country to “burn in” and extrapolate HIV epidemic levels until an assumed future CTx rollout. This process included calibration of ART‐ and disease stage‐specific parameters (online Appendix Table A2) to minimize the difference between the UNAIDS‐reported HIV prevalence, incidence and ART coverage between 2010 and 2021 per country and the respective analogous values in the model. Post‐2021, the model then estimates the diverging (post‐introduction of CTx in year x) trajectories of CTx+ART and ART alone until the year 2100 (the latest available year of US Census international population projections) [19], for a total model time horizon of 90 years. The analysis concerned differences between comparators post‐CTx rollout; prior to this rollout, the trajectories of the CTx+ART and ART alone comparators were equivalent.

A 15‐ to 49‐year‐old population, for which HIV epidemiological statistics are widely available for each included country, entered the model in the year 2010. We did not explicitly model the sex distributions of the populations. Using a decision tree (online Appendix Figure A1), individuals were distributed among health states in the first model cycle according to country‐specific 2010 estimates of HIV prevalence and model‐calibrated parameters. Most individuals entered the model in the uninfected health state; those already HIV positive entered the model with either early HIV (defined as CD4^+^ cell count of >500/μl of blood), chronic HIV (CD4^+^ 200–499/μl) or AIDS (CD4^+^ <200/μl) [20]. Individuals with HIV could be on or off ART based on country‐specific estimates.

In subsequent model cycles, new 15‐year‐olds entered the model (online Appendix Figure A2), a small fraction of whom were HIV positive. Individuals could transition from uninfected to an HIV health state or from one HIV health state to another (Figure 1 and online Appendix Table A3). Individuals automatically transitioned from early HIV infection to chronic HIV after a single semi‐annual model cycle. In the CTx+ART comparator arm, eligible individuals could receive CTx. As determined, however, by the modelled durability of the CTx cure, they could transition to a relapsed HIV health state with costs and disability weights equivalent to chronic HIV until they either received another CTx administration or died. Individuals could transition to death via AIDS‐associated mortality [21, 22] or the background mortality of the modelled country (online Appendix Figure A3). The modelled population size and weighted age composition (online Appendix Figure A4) were based on population projections to the year 2100 derived from the international database tool provided by the US Census Bureau [19]. All individuals entering the model each year were tracked until death or the end of the modelled time horizon.

### 2.2 Parameter calibration

For each country, we implemented a calibration process to derive estimates for unknown model parameters. We utilized an ordinary least squares approach wherein we minimized the sum of the squared residuals among UNAIDS‐reported HIV prevalence, incidence and ART coverage from 2010 to 2021 and the corresponding model‐calculated outputs. Calibrated parameters included those for the levels of disease progression among affected individuals, time until HIV diagnosis, and ART uptake (post‐diagnosis) and re‐uptake (post‐ART discontinuation) rates. Additional information on the calibration process is described in the online Appendix (Table A2).

### 2.3 HIV incidence

The annual probability of HIV infection was dynamically imputed for each semi‐annual model cycle using a constant, country‐specific underlying risk of HIV transmission per person per unit (A) time, multiplied by a time‐varying coefficient (B). The constant transmission risk for each country (A) was estimated from the proportion of individuals in each health state in the first model cycle, their respective relative risk of transmissions and the incidence rate reported for that country in that year by UNAIDS. The dynamic force of infection coefficient was re‐estimated each model cycle from two sub‐components: availability (linearly increasing per year) and efficacy of pre‐exposure prophylaxis (PrEP) and condom usage [7], and the relative risks of transmission according to health state multiplied by the proportion of individuals in each health state. Thus, CTx and ART transition probabilities directly impacted force of infection calculations via the effect they have in moving individuals into health states with lower risk of mortality and of transmission. The relative risk of transmission was highest in early‐stage individuals (26.0x) followed by those with AIDS (7.2x), chronic individuals who have discontinued ART (3.6x), chronic individuals who are on ART (0.08x) and chronic individuals yet to start ART were the reference relative risk (1x) [7, 23, 24]. Uninfected and CTx‐cured individuals were modelled as having a relative transmission risk of zero, while relapsed individuals were assumed to have a relative transmission risk equivalent to being on ART (0.08x). Additional force of infection details are available in online Appendix Table A4.

### 2.4 Curative therapy

Presently, no CTx for HIV exists, and our conceptualization of a future CTx is intended to provide qualitative guidance for interested stakeholders. While we made no assumptions regarding the biological mechanism of the hypothetical CTx, we assumed in all modelled scenarios that it would be administered to age 15+ individuals with HIV infection as a single dose with a one‐time cost, and that successful CTx administrations led to prompt ART‐free suppression of HIV within 6 months. We further assumed that recipients of CTx who have not relapsed: (1) have equivalent quality of health to those in the uninfected population; (2) incur no additional ART cost; (3) are not infectious; (4) cannot be re‐infected upon subsequent exposure to HIV [7]; and (5) that recipients of CTx who have relapsed have the same quality of health and infectiousness as those in the chronic HIV health state.

We also employed a number of variable assumptions for the hypothetical CTx, including (1) a 60% probability of achieving cure; (2) it would be administered only to ART‐adherent individuals (3) who have not yet progressed to AIDS; (4) a year 2030 rollout; (5) a maximum annual uptake rate among eligible individuals of 50% would be reached after (6) 10 years of linear uptake growth; and (7) the median cure duration was 10 years but (8) relapsed individuals would be eligible for repeat administration (Table 1). All eight assumptions were varied according to plausible pessimistic and optimistic values, described below.

**Table 1:** Gene therapy and ART scenario parameters

Curative therapy assumptions	Pessimistic CTx assumptions	Base case CTx assumptions	Optimistic CTx assumptions
Year of availability	2040	2030	2027
Cure probability	30%	60%	90%
Reinfection possibility	Yes	Yes	No
Cure durability (years)	5	10	Lifetime
Eligibility: latest disease stage	Chronic HIV	Chronic HIV	AIDS
Eligibility: patients must be adherent to ART	Yes	Yes	No
Eligibility: repeat CTx administration after relapse	No	Yes	N/A
Maximum uptake proportion/year	25%	50%	75%
Years until maximum uptake achieved	15	10	5

ART assumptions	Pessimistic ART assumptions	Base case ART assumptions	Optimistic ART assumptions
ART uptake/reuptake	−5% per year	Calibrated	+5% per year^a^
ART adherence	−5% per year	SA: 92% [1], US: 82% [1]	+5% per year^a^
ART cost/year	−5% per year	SA: $249 [36], US: $42,000 [37]	+5% per year

Abbreviations: SA, South Africa; US, United States.

^a^ ART uptake (post‐diagnosis), re‐uptake (post‐ART discontinuation) and adherence were rate‐limited to a maximum of 95%.

### 2.5 Other parameters

We calculated semi‐annual transition probabilities for progression from infection to AIDS derived via a median time from infection to AIDS of 14.7 years [23]. The AIDS‐associated mortality probability was calculated from a median time of 8.3 months [21]. We calculated an age‐weighted background mortality transition probability per cycle based on population pyramid projections per year and life tables for each country [19]. The annual costs of ART, PrEP and other HIV standard care services were country‐specific and were obtained via targeted review from multiple published and online data sources (online Appendix Table A1).

The aggregate time spent in each health state was used in conjunction with health state‐specific disability weights to estimate DALYs [15]. DALYs are a measure of population health estimated over the modelled time horizon, calculated here as the sum of the *years of life lost* due to premature mortality (YLLs) from HIV/AIDS and the *years of healthy life lost due to disability* (YLDs) for people living in HIV/AIDS health states [25]. Disability weights for health states were obtained from the Global Burden of Disease study [26].

### 2.6 Modelled countries

Twelve countries were evaluated: France, Germany, Ghana, India, Italy, Kenya, Nigeria, South Africa, Spain, Uganda, the United States and Zambia. For presentational efficiency, we have limited this summary report to South Africa and the United States. For each country, we explored via a set of scenarios the effects of base case, pessimistic and optimistic CTx properties, and in the context of similarly variable properties of ART uptake, ART adherence and ART cost (Table 1).

#### 2.6.1 South Africa

South Africa is an MIC with a high HIV disease burden. The 2010 HIV incidence and prevalence estimates were 15.62 per 1000 [17] and 16.3% [18], respectively, and the model calibrated to 2021 estimates of 7.26 per 1000 and 17.4%, respectively (Figure 2 and online Appendix Figure A5). Calibration resulted in a median time to HIV diagnosis of 0.7 years and that, among those diagnosed, ART coverage was 80% with a 75% ART adherence rate per cycle, and ART uptake post‐diagnosis and re‐uptake post‐discontinuation were 10% and 88% per cycle, respectively (online Appendix Table A2). We used a constant ICER threshold of $3228 per DALY averted for South Africa based on the opportunity cost approach by Ochalek et al. [27].

Figure 2Model predicted HIV prevalence, ART coverage, and AIDS deaths for pre‐ and post‐introduction of gene therapy cure. ART coverage refers to the percentage of adults and children living with HIV currently receiving antiretroviral combination therapy in accordance with the nationally approved treatment protocols (or WHO/UNAIDS standards), antiretroviral regimens prescribed for post exposure prophylaxis are excluded, among the total estimated number of adults and children living with HIV.

**Figure:**
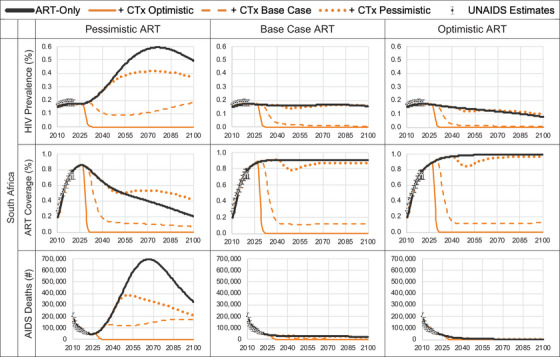

**Figure:**
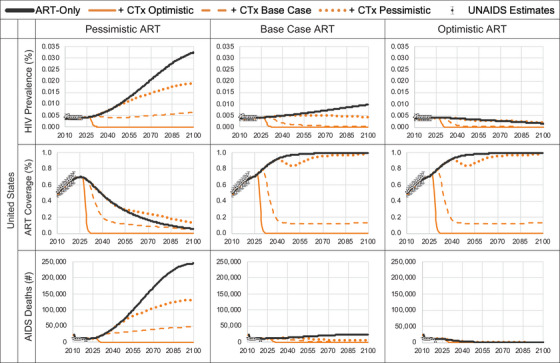

#### 2.6.2 United States

The United States is a high‐income country (HIC) with a low HIV disease burden. The 2010 HIV incidence and prevalence estimates were 0.23 per 1000 [17] and 0.40% [18], respectively, and the model calibrated to 2021 estimates of 0.21 per 1000 and 0.44%, respectively. Calibration resulted in a median time to HIV diagnosis of 0.7 years and that, among those diagnosed, ART coverage was 67% with a 75% ART adherence rate per cycle, and ART uptake post‐diagnosis and re‐uptake post‐discontinuation were 5% and 42% per cycle, respectively. We used a constant cost per DALY averted threshold in the United States of $100,000 [14].

### 2.7 Model analysis

We estimated HIV infections prevented, life years gained, HIV deaths prevented, DALYs averted, DALYs averted per infection prevented and the incremental costs for CTx+ART versus ART alone. Nine scenarios (3 CTx scenarios × 3 ART scenarios) per country were estimated using the values described in Table 1. We held all economic aspects of the model (costs, the discount rate and cost per DALY averted thresholds) constant over the entire 90‐year time horizon, and the base year for future discounting was 2023. Based on the calculated health impacts per country and each country's unique cost‐effectiveness threshold (further details available in the online Appendix pp. 2–3), we then computed the VBP for all 12 countries and examined how the properties of the hypothetical CTx and the base case, pessimistic and optimistic ART programmes per country would affect the VBP. Finally, we measured the uncertainty of our VBP estimates due to intrinsic uncertainties in model parameters with one‐way and probabilistic sensitivity analyses.

## 3 RESULTS

We estimated that the introduction of CTx lowered HIV prevalence and prevented future infections over time, which increased life years, reduced the number of individuals on ART, reduced AIDS‐related deaths, and ultimately led to fewer DALYs versus ART‐alone (Tables 2 and 3, and online Appendix Tables A5–A16). Over time, the reductions in both HIV cases and the numbers on ART led to ART and standard care cost‐savings, with greater cost‐savings (excluding the cost of CTx) in scenarios with greater numbers of cured individuals. While our long‐term projections show that the epidemic would eventually decline without CTx due the effects of (non‐pessimistic) ART and other existing programmes, we found that the introduction of CTx hastens the reductions in numbers living with HIV and the rate of AIDS deaths by decades.

**Table 2:** Curative therapy scenarios and calculation of the value‐based price for South Africa)

Outcomes: CTx+ART versus ART‐only: South Africa	Calculation	Pessimistic CTx, pessimistic ART	Pessimistic CTx, base case ART	Pessimistic CTx, optimistic ART	Base case CTx, pessimistic ART	Base case CTx, base case ART	Base case CTx, optimistic ART	Optimistic CTx, pessimistic ART	Optimistic CTx, base case ART	Optimistic CTx, optimistic ART
Administered CTx doses, 2030–2040		4,521,314	5,595,081	5,387,416	7,145,044	7,506,204	7,522,257	7,200,875	6,593,506	6,443,313
Total infections prevented (difference [%] vs. ART‐only)	A	14,872,894 (−23%)	−144,477 (+1%)	−1,215,392 (+13%)	33,340,279 (−53%)	8,783,006 (−54%)	3,287,400 (−36%)	57,850,634 (−91%)	11,380,510 (−70%)	4,594,543 (−50%)
Infections prevented/1000 person‐years		6.42	−0.03	−0.35	13.06	2.58	0.95	20.60	3.33	1.33
Life years gained/1000^a^ (difference [%] vs. ART‐only)		42,834 (+7.34%)	1695 (+0.24%)	1006 (+0.14%)	96,232 (+11.07%)	7286 (+0.73%)	2282 (+0.23%)	144,577 (+14.95%)	13,767 (+1.25%)	5195 (+0.47%)
Total AIDS deaths averted (difference [%] vs. ART‐only)	B	13,100,643 (−37%)	219,080 (−6%)	80,862 (−4%)	23,151,705 (−66%)	1,533,120 (−39%)	255,349 (−11%)	33,257,944 (−95%)	2,141,208 (−55%)	461,820 (−21%)
AIDS deaths averted/infection prevented	B/A	0.88	−1.52	−0.07	0.69	0.17	0.08	0.57	0.19	0.10
DALYs averted/1000^a^ (difference [%] vs. ART‐only)	C	55,611 (−8.02%)	−4000 (+0.55%)	−4788 (+0.66%)	148,987 (−14.27%)	20,192 (−1.97%)	11,340 (−1.11%)	223,637 (−18.76%)	32,146 (−2.84%)	18,206 (−1.62%)
DALYs averted/infection prevented^a^	C/A	3.7	27.7	3.9	4.5	2.3	3.4	3.9	2.8	4.0
Incremental HIV^†^ cost/1000 (difference [%] vs. ART‐only)	D	−$55.5 M (−10.4%)	−$6.2 M (−2.6%)	$2.9 M (+1.4%)	−$335.5 M (−50.7%)	−$170.9 M (−50.8%)	−$131.6 M (−43.3%)	−$558.2 M (−80.1%)	−$240.1 M (−64.8%)	−$187.3 M (−55.6%)
Incremental ART cost/1000^a^ (difference [%] vs. ART‐only)	E	$141 M (+306%)	−$2 M (−7%)	$ M (−6%)	−$29 M (−50%)	−$27 M (−78%)	−$6 M (−63%)	−$60 M (−96%)	−$36 M (−94%)	−$11 M (−87%)
*Value‐based price/patient: curative therapy*		$19,977	$0^b^	$0^b^	$40,867	$14,961	$10,460	$197,611	$60,939	$42,208
Incremental CTx cost/1000 (using VBP)^a^	F	$94 M	−$5 M	−$18 M	$846 M	$263 M	$175 M	$1340 M	$380 M	$257 M
Incremental HIV+ART+CTx cost/1000^a^ (difference [%] vs. ART‐only)	D+E+F	$180 M (+31.0%)	−$13 M (−4.9%)	−$15 M (−7.2%)	$481 M (+66.8%)	$65 M (+17.6%)	$37 M (+11.7%)	$722 M (+95.1%)	$104 M (+25.4%)	$59 M (+16.8%)
Total cost per DALY averted (difference [%] vs. ART‐only)	(D+E+F)/C	$3228	$3228	$3228	$3228	$3228	$3228	$3228	$3228	$3228

^a^ Calculated using a discount rate of 3% per year. All outcomes are calculated over a 90‐year time horizon from 2010 until 2100.

^b^ A zero value‐based price is assigned in lieu of negative value‐based price due to increased HIV healthcare costs and/or worse DALYs avoided.

**Table 3:** Curative therapy scenarios and calculation of the value‐based price for the United States

Outcomes: CTx+ART versus ART‐only: United States	Calculation	Pessimistic CTx, pessimistic ART	Pessimistic CTx, base case ART	Pessimistic CTx, optimistic ART	Base case CTx, pessimistic ART	Base case CTx, base case ART	Base case CTx, optimistic ART	Optimistic CTx, pessimistic ART	Optimistic CTx, base case ART	Optimistic CTx, optimistic ART
Administered CTx doses, 2030–2040		258,057	617,582	590,350	550,116	778,123	828,582	913,337	855,718	803,706
Total infections prevented (difference [%] vs. ART‐only)	A	7,635,726 (−35%)	2,380,445 (−45%)	−118,395 (+10%)	15,488,840 (−72%)	3,927,605 (−74%)	304,908 (−27%)	20,798,870 (−96%)	4,619,402 (−87%)	512,644 (−45%)
Infections prevented/1000 person‐years		0.44	0.14	−0.01	0.89	0.22	0.02	1.19	0.26	0.03
Life years gained/1000^a^ (difference [%] vs. ART‐only)		8048 (+0.23%)	1998 (+0.06%)	109 (+0.00%)	20,568 (+0.42%)	4039 (+0.08%)	326 (+0.01%)	33,473 (+0.62%)	7047 (+0.13%)	1391 (+0.03%)
Total AIDS deaths averted (difference [%] vs. ART‐only)	B	3,603,686 (−35%)	730,970 (−43%)	8224 (−2%)	7,324,644 (−71%)	1,137,384 (−66%)	35,935 (−9%)	9,978,114 (−97%)	1,423,882 (−83%)	112,312 (−29%)
AIDS deaths averted/infection prevented	B/A	0.47	0.31	−0.07	0.47	0.29	0.12	0.48	0.31	0.22
DALYs averted/1000^a^ (difference [%] vs. ART‐only)	C	12,177 (−0.35%)	2666 (−0.08%)	−415 (+0.01%)	31,962 (−0.65%)	7483 (−0.15%)	1346 (−0.03%)	50,836 (−0.94%)	11,946 (−0.22%)	3096 (−0.06%)
DALYs averted/infection prevented^a^	C/A	1.6	1.1	3.5	2.1	1.9	4.4	2.4	2.6	6.0
Incremental HIV^†^ cost/1000 (difference [%] vs. ART‐only)	D	−$393.1 M (−21.6%)	−$158.1 M (−16.4%)	$9.7 M (+0.4%)	−$1103.8 M (−52.1%)	−$457.0 M (−36.1%)	−$147.3 M (−5.2%)	−$1701.2 M (−76.8%)	−$632.0 M (−46.3%)	−$232.2 M (−7.9%)
Incremental ART cost/1000^a^ (difference [%] vs. ART‐only)	E	$2140 M (+428%)	−$60 M (−11%)	−$5 M (−6%)	−$139 M (−21%)	−$568 M (−79%)	−$117 M (−61%)	−$670 M (−95%)	−$741 M (−96%)	−$207 M (−87%)
*Value‐based price/patient: curative therapy*		$0^b^	$786,019	$0^b^	$2,419,892	$812,280	$203,667	$8,702,245	$3,190,277	$987,563
Incremental CTx cost/1000 (using VBP)^a^	F	−$529 M	$485 M	−$46 M	$4439 M	$1774 M	$399 M	$7454 M	$2567 M	$748 M
Incremental HIV+ART+CTx cost/1000^a^ (difference [%] vs. ART‐only)	D+E+F	$1218 M (+52.6%)	$267 M (+17.4%)	−$42 M (−1.6%)	$3196 M (+115.3%)	$748 M (+37.6%)	$135 M (+4.5%)	$5084 M (+174.3%)	$1195 M (+55.9%)	$310 M (+9.8%)
Total cost per DALY averted (difference [%] vs. ART‐only)	(D+E+F)/C	$100,000	$100,000	$100,000	$100,000	$100,000	$100,000	$100,000	$100,000	$100,000

^a^ Calculated using a discount rate of 3% per year.

^b^ A zero value‐based price is assigned in lieu of negative value‐based price due to increased HIV healthcare costs and/or worse DALYs avoided. All outcomes are calculated over a 90‐year time horizon from 2010 until 2100.

The VBP estimates for each of the 12 included countries, under each combination of assumptions for CTx and ART programmes, are shown in Table 4. Within each country, the VBP was higher when the efficacy and rollout of CTx were more favourable and ART uptake, adherence and cost were less favourable. Some scenario combinations (20 of 108 total) resulted in CTx leading to increased HIV healthcare costs, worse DALYs avoided or both, resulting in a negative VBP (reported as $0).

**Table 4:** Value‐based price of curative therapy per modelled country

Country	Pessimistic CTx, pessimistic ART	Pessimistic CTx, base case ART	Pessimistic CTx, optimistic ART	Base case CTx, pessimistic ART	Base case CTx, base case ART	Base case CTx, optimistic ART	Optimistic CTx, pessimistic ART	Optimistic CTx, base case ART	Optimistic CTx, optimistic ART
Ghana	$24,950	$12,504	$0^a^	$32,513	$12,166	$3241	$117,415	$53,309	$17,104
India	$0^a^	$2202	$0^a^	$6406	$6378	$2787	$27,289	$22,713	$11,515
Kenya	$0^a^	$363	$0^a^	$26,448	$5417	$2808	$107,939	$22,113	$12,250
Nigeria	$0^a^	$5899	$0^a^	$16,583	$6808	$2092	$69,733	$28,925	$9542
South Africa	$19,977	$0^a^	$0^a^	$40,867	$14,961	$10,460	$197,611	$60,939	$42,208
Uganda	$629	$2828	$0^a^	$15,016	$5616	$2023	$113,594	$26,065	$9353
Zambia	$0^a^	$3345	$0^a^	$21,186	$8276	$3109	$112,140	$34,787	$13,767
France	$0^a^	$657,324	$0^a^	$1,547,065	$513,293	$118,433	$9,217,161	$2,467,945	$643,335
Germany	$0^a^	$236,214	$0^a^	$741,369	$310,920	$124,864	$2,721,230	$1,070,909	$477,901
Italy	$370,721	$21,647	$0^a^	$490,468	$102,357	$59,981	$1,360,842	$338,009	$211,571
Spain	$121,863	$91,163	$0^a^	$439,660	$134,977	$53,851	$1,651,333	$489,086	$213,642
United States	$0^a^	$786,019	$0^a^	$2,419,892	$812,280	$203,667	$8,702,245	$3,190,277	$987,563

^a^ A zero value‐based price is assigned in lieu of negative value‐based price due to increased HIV healthcare costs and/or worse DALYs avoided.

For South Africa, the (non‐negative) VBP ranged from $10,500 (optimistic ART, base case CTx, rounded) to $197,600 (pessimistic ART, optimistic CTx, rounded), while the VBP in the United States ranged from $203,700 (optimistic ART, base CTx, rounded) to $8.7 million (pessimistic ART, optimistic CTx, rounded). The VBP also notably varied among countries, with higher VBPs estimated for countries where the HIV caseload was greater and in countries with greater income (and so, it is assumed, greater per capita spending on health).

**Figure 3:**
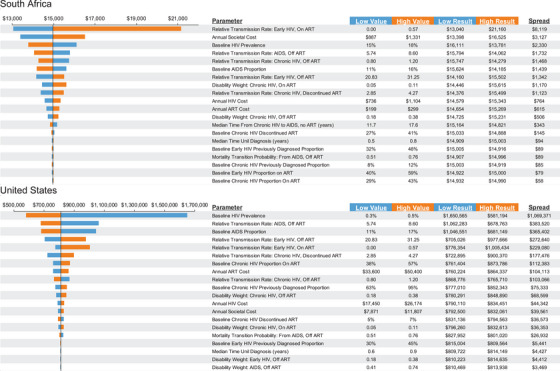
One‐way sensitivity analyses of value‐based price: top 20 most influential parameters. In one‐way sensitivity analysis, one parameter at a time is varied to its plausible low and high values while keeping all other parameters constant.

We found that the range in VBP induced for South Africa was −13% to +41% of the VBP estimated under the base case (Figure 3), with the HIV transmission rate among individuals with early HIV having the greatest impact. For the United States, the range was −28% to +208%, with the HIV prevalence in 2010 being the most impactful parameter. This wide variability in the VBA was supported by the results of the probabilistic analysis (online Appendix Table A17).

## 4 DISCUSSION

We investigated the potential health impacts of a future CTx intervention for HIV leading to durable, ART‐free remission (“cure”) in individuals and declines in HIV prevalence in individual countries. The cost‐savings and health benefits conferred by CTx decreasing the risk of AIDS and the cost of ART were used to calculate the VBP for CTx for each country that, given its spending on healthcare, could be willing to pay per CTx administration. We found that the health benefits of CTx were greatest in circumstances where the HIV burden is higher (e.g. South Africa) and when making “pessimistic” assumptions for ART, including lower ART uptake and adherence. We also found that higher calculated VBPs were associated with greater CTx‐conferred health benefits.

Previous modelling analyses have delved into questions pertaining to the impact of a future HIV cure. Beacroft and Hallett similarly found that an HIV cure would have the greatest impact in geographies where the epidemic is less well‐controlled [7]. They additionally found that targeting those most likely to transmit the virus (early‐stage HIV) and maximizing the duration of viral suppression could increase impact. Phillips et al. estimated that a cure would result in >0.53 million DALYs averted over 2022–2042 in sub‐Saharan Africa, with a reduction in HIV programme costs of $300 million (−8.7%) and estimated that a VBP up to $1400 would be justified [8]. This is lower than in our analysis, which differs primarily in that we assumed a CTx rollout beginning in 2030 instead of 2022 and that we modelled outcomes to the year 2100. Dimitrov et al. estimated a cure's impact from the perspective of eligibility based on ART adherence and found that HIV incidence would not be reduced unless the intervention was available to all individuals regardless of adherence [28]. We similarly found that the beneficial impact of an HIV cure is enhanced if there are fewer eligibility restrictions, although it may be more efficient to target those with the highest transmission risks.

Achieving universal access to CTx for HIV will be challenging. The potential for high manufacturing and delivery costs, as well as a potential lack of competition among manufacturers, mean there will be barriers to equitable pricing in a real‐world setting [29, 30, 31, 32]. Determining the long‐term value of a high‐priced CTx is also challenging in a world in which pricing mechanisms often focus on short‐term budget impact analyses that may undervalue longer‐term health benefits to individual patients [13]. We contend that pharmaceutical manufacturers should tailor prices based on the willingness of countries to pay for health benefits accrued over a lifetime and that setting a target threshold at this early phase of thinking about CTx for HIV is a crucial step in ensuring widespread access and uptake. High‐priced, one‐time, upfront payments are likely to put universal access and affordability out of reach in most LMICs; therefore, instalment payment options could be explored [13, 29, 31]. Furthermore, performance‐based reimbursement schemes, wherein coverage and reimbursement are linked to real‐world, long‐term efficacy, might offer a way to reduce the price paid for CTx if its health impact is less than initially anticipated [33, 34].

Our study has limitations that warrant mention. First, our estimates are highly contingent on assumptions about the future uptake of ART and the efficacy, durability, eligibility and uptake of a hypothetical CTx, among others. Therefore, we suggest that our results be understood as exploratory in nature and that they should be continually reassessed as new information becomes available. Second, it was challenging to identify country‐specific estimates beyond HIV incidence and prevalence for all countries; these data gaps include treatment‐specific costs, the distribution of patients across early/chronic/AIDS stages, and the uptake and adherence to ART, the latter two categories ultimately requiring parameter calibration. Regional‐based assumptions were made for some countries in the calculation of their health outcomes and VBP. Third, we did not include barriers to treatment access in LMICs other than their limited abilities to pay for future CTx; in reality, LMICs contend with a much lower density of health workers and hospital beds per population compared to HICs, and LMICs have a larger proportion of out‐of‐pocket expenses, among other factors that could be barriers to the use of CTx [35]. Fourth, while much remains unknown about the effectiveness and durability of CTx, it likely will not reverse the systematic impact of HIV on long‐term health, and may also lead to disengagement with other supportive HIV care; therefore, our assumption that a cure leads to health equivalent to the uninfected population likely inflates the VBP and this should be explored in future analyses. Fifth, our analysis makes no assumptions regarding the mechanism of curative therapy other than it being a one‐time administration that either succeeds or fails, which may greatly oversimplify the eventual clinical application. Lastly, we did not explore in depth the intricacies of manufacturing and distributing a future CTx but, rather, estimated its VBP under various epidemic and economic circumstances. Individual manufacturers will need to evaluate whether their combined operations can meet the price per CTx administration proposed in this study.

## 5 CONCLUSIONS

We demonstrate here that a cost of CTx in the thousands for LMICs, and 10‐fold greater than that in the United States, would be consistent with it being cost‐effective for those countries to use. The DALYs averted and cost‐savings conferred by CTx increased with more optimistic scenarios for CTx and more pessimistic scenarios for ART. With greater CTx cure probability, durability and scale up, CTx commands a higher VBP, while improvements in ART uptake and adherence may mitigate its value. Our framework can be utilized for estimating this price given a wide range of potential CTx impact and HIV epidemiological scenarios. If these manufacturers can allow for the future prices of CTx to be within these envelopes, then the potential of CTx may be unlocked and the end of AIDS could come one step closer.

## COMPETING INTERESTS

GFG received funding for this research from the Bill & Melinda Gates Foundation, Seattle, USA. TBH received funding from HCD Economics, Daresbury, UK.

## AUTHORS’ CONTRIBUTIONS

GFG and TBH: Conceptualization.

GFG and TBH: Data curation.

GFG: Formal analysis.

GFG: Funding acquisition.

GFG and TBH: Investigation.

GFG and TBH: Methodology.

GFG: Project administration.

GFG: Resources.

GFG: Software.

GFG: Supervision.

GFG and TBH: Validation.

GFG: Visualization.

GFG: Writing—original draft.

GFG and TBH: Writing—review & editing.

## FUNDING

This research was funded by the Bill & Melinda Gates Foundation.

## Supporting information


**Supporting information**. Additional methods and results can be found in the supplementary appendix.
Modelling overview and calculation of the value‐based price
**Table A1**. Model parameters: assumptions and literature‐derived
**Table A2**. Calibrated model parameters
**Figure A1**. Decision tree for population distribution at cycle zero.
**Table A3**. Transition probability matrix for the open cohort model
**Table A4**. Calculation of the force of HIV infection per semi‐annual model cycle
**Figure A2**. Model input: country‐specific average population age per model cycle over an 80‐year time horizon.
**Figure A3**. Model input: country‐specific, age‐weighted background mortality per model cycle over an 80‐year time horizon.
**Figure A4**. Model input: number of 15‐year‐olds entering the open cohort model per year, by country.
**Figure A5**. Model predicted HIV prevalence pre‐ and post‐introduction of curative therapy cure.
**Tables A5–A16**. Model results for each included country
**Table A17**. Results of probabilistic sensitivity analysis: curative therapy versus ART (base case for both comparators)

## Data Availability

The model and its supporting data are openly available here.
