# Supplementary material for: The long‐term impact and value of curative therapy for HIV: a modelling analysis
Source: J Int AIDS Soc. 2023 Sep 25;26(9):e26170. doi: 10.1002/jia2.26170 (PMC10519941; doi:10.1002/jia2.26170)
Supplement: Supplementary file 1 — Supporting information. Additional methods and results can be found in the supplementary appendix. Modelling overview and calculation of the value‐based price Table A1. Model parameters: assumptions and literature‐derived Table A2. Calibrated model parameters Figure A1. Decision tree for population distribution at cycle zero. Table A3. Transition probability matrix for the open cohort model Table A4. Calculation of the force of HIV infection per semi‐annual model cycle Figure A2. Model input: country‐specific average population age per model cycle over an 80‐year time horizon. Figure A3. Model input: country‐specific, age‐weighted background mortality per model cycle over an 80‐year time horizon. Figure A4. Model input: number of 15‐year‐olds entering the open cohort model per year, by country. Figure A5. Model predicted HIV prevalence pre‐ and post‐introduction of curative therapy cure. Tables A5–A16. Model results for each included country Table A17. Results of probabilistic sensitivity analysis: curative therapy versus ART (base case for both comparators) [file JIA2-26-e26170-s001.docx]

**Supplementary Appendix:**

**The Long-Term Impact and Value of Curative Therapy for HIV:**

**A Modelling Analysis**

Gregory F. Guzauskas^1,2^ and Timothy B. Hallett^3^

1. The Comparative Health Outcomes, Policy, and Economics Institute, University of Washington, Seattle, WA, USA
2. HCD Economics, Daresbury, UK
3. MRC Centre for Global Infectious Disease Analysis, Imperial College London, London, UK

Contents:

Modelling Overview and Calculation of the Value-Based Price

Table A1. Model Parameters: Assumptions and Literature-Derived

Table A2. Calibrated Model Parameters

Figure A1. Decision tree for population distribution at cycle zero.

Table A3. Transition probability matrix for the open cohort model.

Table A4. Calculation of the force of HIV infection per semiannual model cycle.

Figure A2. Model Input: country-specific average population age per model cycle over an 80-year time horizon.

Figure A3. Model Input: country-specific, age-weighted background mortality per model cycle over an 80-year time horizon.

Figure A4. Model Input: Number of 15-Year-Olds Entering the Open Cohort Model per Year, by Country

Figure A5. Model Predicted HIV Prevalence Pre- and Post-Introduction of Curative Therapy Cure

Tables A5-A16. Model Results for Each Included Country

Table A17. Results of probabilistic sensitivity analysis: Curative Therapy versus ART (base case for both comparators)

*Modelling Overview*

Decision-analytic state-transition modelling is a common health economic evaluation approach that can be applied to different populations and diseases.^1,2^ Modelled individuals or proportions of modelled cohorts reside within “health states” that correspond to a specific stage of disease natural history for a given time period. From a specific health state (other than dead), individuals can “transition” to different health states over time; the likelihood of such moves for a particular time period is called a “transition probability”.^2^

The time spent in each health state can be used in conjunction with state values (e.g., life-years, health state-specific disability weights, and costs) to estimate life expectancy, disability-adjusted life years (DALYs), and expected costs.^2^ DALYs are the measure of population health estimated over the modelled time horizon, calculated here as the sum of the *years of life lost* due to premature mortality (YLLs) from HIV/AIDS and the *years of healthy life lost due to disability* (YLDs) for people living in HIV/AIDS health states.^3^ To compute the YLD, the number of people in each health state per model cycle is multiplied by a disability weight, where a value of 0 represents perfect health, 1 represents dead, and values for states of health in between represent degrees of morbidity.

The ICER is calculated as the difference in cost between modelled comparators divided by the difference in effectiveness (DALYs in this case); i.e., it is the cost per DALY averted. We assume that for a new intervention to be “cost-effective”, the DALYs averted by the intervention should exceed some threshold generated using those same resources in a different way in a particular country. Put another way, the “cost-effectiveness threshold” reflects the country-specific benchmark for the reduction of DALYs per unit cost that a new intervention must exceed to be “cost-effective”.^1^ Following the approach of Mohan et al.,^4^ knowing these thresholds allows for the calculation of the VBP, which is the maximum cost for an intervention with a given health benefit that is consistent with the new intervention meeting this criterion. In our model we have used the accepted ICER thresholds for the high-income countries (HICs) that regularly employ them and a study by Ochalek et al.^5^ that provides a framework for generating LMIC-specific threshold estimates.

*Calculation of the VBP*

With t_DALYs = (total DALYs for the comparator), t_cost = (total overall cost for the comparator), cost_CTx = (the cost of gene therapy), cost_CTx_ART = (the cost of ART in the CTx comparator), cost_CTx_standard_care = the cost of standard HIV care in the CTx comparator), cost_CTx_societal = (societal costs in the CTx comparator), t_cost_CTx = (cost_CTx + cost_CTx_ART + cost_CTx_standard_care + cost_CTx_societal), and ICER = (the country-specific cost-effectiveness threshold), the VBP of CTx is calculated as follows:

If

[t_cost_CTx – t_cost_ART] ÷ [t_DALYs_ART – t_DALYs_CTx] = ICER,

Then

VBP = cost_CTx = [t_DALYs_ART – t_DALYs_CTx] * ICER – t_cost_ART – [cost_CTx_ART – cost_CTx_standard_care – cost_CTx_societal].

**Table A1. Model Parameters: Assumptions and Literature-Derived**

| Parameter | Default | < Sensitivity Range > | | SE | PSA Distribution | Variable Name | Source |
| --- | --- | --- | --- | --- | --- | --- | --- |
|  |  |  |  |  |  |  |  |
| Curative Therapy (CTx) Parameters |  |  |  |  |  |  |  |
| Year of Curative Therapy Availability | 2030 | 2027 | 2040 |  |  | year_ctx | Assumption |
| Cure Probability | 60% | 30% | 90% | 0.15 | Beta | ctx_cure_rate | Assumption |
| Relapse Possibility | TRUE | TRUE | FALSE |  |  | ctx_relapse | Assumption |
| └ Median Cure Duration (years) | 10 | 8 | 12 | 1.02 | Normal |  | Assumption |
| └ Conversion to Semiannual Probability | 0.034 | 0.042 | 0.028 |  |  | ctx_cure_dur | Calculated |
| Maximum Stage Eligibility for Curative Therapy | Chronic | Acute | AIDS |  |  |  | Assumption |
| Treatment Status Eligibility for Curative Therapy | On ART Only | On ART Only | Off ART Only |  |  |  | Assumption |
| Repeat Curative Therapy After Relapse/Failure? | TRUE | TRUE | FALSE |  |  | ctx_repeat | Assumption |
| Maximum Annual Curative Therapy Uptake | 50% | 25% | 75% | 0.13 | Beta | ctx_uptake | Assumption |
| └ Ramp Up Time to Maximum Uptake (years) | 10 | 5 | 15 | 2.55 | Normal | ctx_ramp | Assumption |
|  |  |  |  |  |  |  |  |
| Relative Rate of Transmitting HIV by Health State |  |  |  |  |  |  |  |
| Uninfected/Cured | 0 |  |  |  |  | transm_cured | Assumption |
| *HIV+, With ART* |  |  |  |  |  |  |  |
| Acute | 0.08 | 0.001 | 0.57 | 1.62 | LogNormal | transm_art_acute | Donnell et al. ^6^ |
| Chronic | 0.08 | 0.001 | 0.57 | 1.62 | LogNormal | transm_art_chronic | Donnell et al. ^6^ |
| *HIV+, Without ART* |  |  |  |  |  |  |  |
| Acute | 26.04 | 20.83 | 31.25 | 0.1 | LogNormal | transm_noart_acute | Hollingsworth et al. ^7^ |
| Chronic | 1 | 0.8 | 1.2 | 0.1 | LogNormal | transm_noart_chronic | Hollingsworth et al. ^7^ |
| AIDS | 7.17 | 5.74 | 8.6 | 0.1 | LogNormal | transm_noart_aids | Hollingsworth et al. ^7^ |
| Discontinued ART | 3.56 | 2.85 | 4.27 | 0.1 | LogNormal | transm_noart_disc | Beacroft et al. ^8^ |
|  |  |  |  |  |  |  |  |
|  |  |  |  |  |  |  |  |
| HIV Disability Weight Parameters |  |  |  |  |  |  |  |
| Uninfected / Cured via Curative Therapy | 0 |  |  |  |  | dis_healthy | Assumption |
| *HIV+, With ART* |  |  |  |  |  |  |  |
| Acute HIV | 0.08 | 0.05 | 0.11 | 0.02 | Beta | dis_art_acute | Saloman et al. ^9^ |
| Chronic HIV | 0.08 | 0.05 | 0.11 | 0.02 | Beta | dis_art_chronic | Saloman et al. ^9^ |
| AIDS | 0.58 | 0.41 | 0.74 | 0.09 | Beta | dis_art_aids | Saloman et al. ^9^ |
| *HIV+, Without ART* |  |  |  |  |  |  |  |
| Acute HIV | 0.27 | 0.18 | 0.38 | 0.05 | Beta | dis_noart_acute | Saloman et al. ^9^ |
| Chronic HIV | 0.27 | 0.18 | 0.38 | 0.05 | Beta | dis_noart_chronic | Saloman et al. ^9^ |
| AIDS | 0.58 | 0.41 | 0.74 | 0.09 | Beta | dis_noart_aids | Saloman et al. ^9^ |
|  |  |  |  |  |  |  |  |
| Modeled Costs |  |  |  |  |  |  |  |
| Annual ART Cost |  |  |  |  |  | cost_art |  |
| Ghana | $225 | $180 | $270 | $23 | Normal |  | Mikkelsen et al. ^10^ |
| India | $738 | $590 | $886 | $75 | Normal |  | Sharma et al. ^11^ |
| Kenya | $300 | $240 | $360 | $31 | Normal |  | CDC & Kenya Ministry of Health ^12^ |
| Nigeria | $231 | $185 | $277 | $24 | Normal |  | Bautista-Arredondo et al. ^13^ |
| South Africa | $249 | $199 | $299 | $25 | Normal |  | Meyer-Rath et al. ^14^ |
| Uganda | $76 | $61 | $91 | $8 | Normal |  | Moreland et al. ^15^ |
| Zambia | $356 | $285 | $427 | $36 | Normal |  | Tucker et al. ^16^ |
| France | $14,710 | $11,768 | $17,652 | $1,501 | Normal |  | ECDC ^17^ |
| Germany | $22,861 | $18,288 | $27,433 | $2,333 | Normal |  | ECDC ^17^ |
| Italy | $8,445 | $6,756 | $10,134 | $862 | Normal |  | ECDC ^17^ |
| Spain | $7,510 | $6,008 | $9,012 | $766 | Normal |  | ECDC ^17^ |
| USA | $42,000 | $33,600 | $50,400 | $4,286 | Normal |  | McCann et al. ^18^ |
| Annual PrEP Cost |  |  |  |  |  | cost_prep |  |
| Ghana | $304 | $243 | $365 | $31 | Normal |  | Assumption (=Nigeria) |
| India | $113 | $90 | $136 | $12 | Normal |  | Ten Brink et al. ^19^ |
| Kenya | $131 | $104 | $157 | $13 | Normal |  | Wanga et al. ^20^ |
| Nigeria | $304 | $243 | $365 | $31 | Normal |  | UNAIDS ^21^ |
| South Africa | $145 | $116 | $174 | $15 | Normal |  | van Vliet et al. ^22^ |
| Uganda | $199 | $159 | $238 | $20 | Normal |  | Ying et al. ^23^ |
| Zambia | $458 | $367 | $550 | $47 | Normal |  | Hendrickson et al. ^24^ |
| France | $3,940 | $3,152 | $4,727 | $402 | Normal |  | Durand-Zelaski et al. ^25^ |
| Germany | $1,006 | $805 | $1,207 | $103 | Normal |  | Marcus et al. ^26^ |
| Italy | $1,510 | $1,208 | $1,812 | $154 | Normal |  | IrsiCaxia ^27^ |
| Spain | $1,510 | $1,208 | $1,812 | $154 | Normal |  | IrsiCaxia ^27^ |
| USA | $2,276 | $1,821 | $2,731 | $232 | Normal |  | Kay et al. ^28^ |
| Annual HIV Cost |  |  |  |  |  | cost_hiv |  |
| Ghana | $734 | $587 | $880 | $75 | Normal |  | Rosen et al. ^29^ |
| India | $436 | $349 | $523 | $44 | Normal |  | Vassall et al. ^30^ |
| Kenya | $734 | $587 | $880 | $75 | Normal |  | Assumption (=Ghana) |
| Nigeria | $140 | $112 | $168 | $14 | Normal |  | Durosinmi-Etti et al. ^31^ |
| South Africa | $920 | $736 | $1,104 | $94 | Normal |  | Tagar et al. ^32^ |
| Uganda | $325 | $260 | $390 | $33 | Normal |  | Moreland et al. ^15^ |
| Zambia | $375 | $300 | $450 | $38 | Normal |  | Tagar et al. ^32^ |
| France | $12,373 | $9,898 | $14,848 | $1,263 | Normal |  | Columbie et al. ^33^ |
| Germany | $10,360 | $8,288 | $12,432 | $1,057 | Normal |  | Wolf et al. ^34^ |
| Italy | $11,458 | $9,166 | $13,749 | $1,169 | Normal |  | Taramasso et al. ^35^ |
| Spain | $8,680 | $6,944 | $10,416 | $886 | Normal |  | Lopez-Bastida et al. ^36^ |
| USA | $21,812 | $17,450 | $26,174 | $2,226 | Normal |  | CDC ^37^ |
| Annual Societal Cost |  |  |  |  |  | cost_soc |  |
| Ghana | $242 | $194 | $290 | $25 | Normal |  | GBD Health Financing Network ^38^ |
| India | $236 | $189 | $283 | $24 | Normal |  | GBD Health Financing Network ^38^ |
| Kenya | $187 | $150 | $224 | $19 | Normal |  | GBD Health Financing Network ^38^ |
| Nigeria | $216 | $173 | $259 | $22 | Normal |  | GBD Health Financing Network ^38^ |
| South Africa | $1,109 | $887 | $1,331 | $113 | Normal |  | GBD Health Financing Network ^38^ |
| Uganda | $159 | $127 | $191 | $16 | Normal |  | GBD Health Financing Network ^38^ |
| Zambia | $241 | $193 | $289 | $25 | Normal |  | GBD Health Financing Network ^38^ |
| France | $4,741 | $3,793 | $5,689 | $484 | Normal |  | GBD Health Financing Network ^38^ |
| Germany | $5,532 | $4,426 | $6,638 | $565 | Normal |  | GBD Health Financing Network ^38^ |
| Italy | $3,445 | $2,756 | $4,134 | $352 | Normal |  | GBD Health Financing Network ^38^ |
| Spain | $3,363 | $2,690 | $4,036 | $343 | Normal |  | GBD Health Financing Network ^38^ |
| USA | $9,839 | $7,871 | $11,807 | $1,004 | Normal |  | GBD Health Financing Network ^38^ |

**Table A2. Calibrated Model Parameters**

| Variable name: | art_uptake | art_adh_chronic | art_reuptake | aids_0 | offart_chronic | preart_aids | diag_chronic | onart_early | onart_chronic | diag_time |
| --- | --- | --- | --- | --- | --- | --- | --- | --- | --- | --- |
|  | Post-Diagnosis ART Uptake Probability/ Cycle | Treatment Adherence Once on ART | ART Re-Uptake Post-Discontinuation | Proportion of AIDS Individuals Pre-ART in Cycle 0* | Proportion Discontinued ART in Cycle 0 | Proportion of Infected Individuals with AIDS in Cycle 0 | Proportion of Previously Diagnosed Individuals in Cycle 0 | Proportion of Early HIV Individuals on ART in Cycle 0 | Proportion of Chronic HIV Individuals on ART in Cycle 0 | Median Time Until Diagnosis (years) |
| Ghana | 4% | 75% | 95% | 73% | 5% | 15% | 30% | 50% | 5% | 0.84 |
| **India** | 5% | 75% | 49% | 150% | 18% | 10% | 28% | 50% | 19% | 0.82 |
| **Kenya** | 10% | 90% | 95% | 59% | 21% | 5% | 34% | 50% | 9% | 0.79 |
| **Nigeria** | 9% | 75% | 95% | 76% | 10% | 14% | 5% | 46% | 5% | 2.43 |
| **South Africa** | 10% | 75% | 88% | 63% | 34% | 13% | 10% | 50% | 36% | 0.67 |
| **Uganda** | 15% | 75% | 95% | 77% | 5% | 13% | 5% | 38% | 5% | 1.15 |
| **Zambia** | 15% | 75% | 95% | 62% | 33% | 11% | 5% | 45% | 14% | 2.06 |
| **France** | 13% | 75% | 14% | 53% | 32% | 14% | 82% | 49% | 50% | 0.74 |
| **Germany** | 26% | 75% | 9% | 40% | 43% | 15% | 89% | 48% | 52% | 0.84 |
| **Italy** | 12% | 75% | 95% | 103% | 5% | 11% | 57% | 50% | 72% | 1.26 |
| **Spain** | 15% | 88% | 23% | 44% | 5% | 12% | 62% | 39% | 66% | 1.73 |
| **USA** | 5% | 75% | 42% | 61% | 6% | 14% | 79% | 50% | 48% | 0.74 |

*Values used in cycle 0 (year 2010) parameters are for model “burn-in” purposes and may reflect irrational values. Proportions in health states stabilize prior to year 2023. QALY and cost outcomes are not estimated in the model prior to year 2023.

**Figure A1. Decision tree for population distribution at cycle zero.**

**Table A3. Transition probability matrix for the open cohort model.**

|  |  | From Health State → | | | | | | | | | | | | |
| --- | --- | --- | --- | --- | --- | --- | --- | --- | --- | --- | --- | --- | --- | --- |
| To Health State → | **Incoming**  **15-year-olds** | **Uninfected** | **Early HIV, Unaware** | **Early HIV, Aware** | **Progressive HIV, Unaware** | **Progressive HIV, Aware** | **AIDS, Aware** | **Early HIV, On ART** | **Progressive HIV, On ART** | **Progressive HIV, Off ART** | **AIDS, Off ART** | **Cured via CTx** | **Relapsed/Reinfected** | **Dead** |
|  | **Uninfected** | remainder | inc_c*(1-diag_acute) | inc_c*diag_acute*(1-art_uptake) |  |  |  | inc_c*diag_acute*art_uptake |  |  |  |  |  | b_mort_c |
|  | **Early HIV, Unaware** |  |  |  | remainder |  |  |  |  |  |  |  |  | b_mort_c |
|  | **Early HIV, Aware** |  |  |  |  | remainder |  |  | art_uptake |  |  | ctx_cure_rate*ctx_uptake |  | b_mort_c |
|  | **Progressive HIV, Unaware** |  |  |  | remainder | diag_time | noart_chronic_aids |  |  |  |  |  |  | b_mort_c |
|  | **Progressive HIV, Aware** |  |  |  |  | remainder | noart_chronic_aids |  | art_uptake |  |  | ctx_cure_rate*ctx_uptake |  | b_mort_c |
|  | **AIDS, Aware** |  |  |  |  |  | remainder |  | art_uptake |  |  | ctx_cure_rate*ctx_uptake |  | max(mort_noart_aids, b_mort_c) |
|  | **Early HIV, On ART** |  |  |  |  |  |  |  | remainder |  |  | ctx_cure_rate*ctx_uptake |  | b_mort_c |
|  | **Progressive HIV, On ART** |  |  |  |  |  |  |  | remainder | 1-art_adh_chronic |  | ctx_cure_rate*ctx_uptake |  | b_mort_c |
|  | **Progressive HIV, Off ART** |  |  |  |  |  |  |  | art_reuptake | remainder | noart_chronic_aids | ctx_cure_rate*ctx_uptake |  | b_mort_c |
|  | **AIDS, Off ART** |  |  |  |  |  |  |  | art_reuptake |  | remainder | ctx_cure_rate*ctx_uptake |  | max(mort_noart_aids, b_mort_c) |
|  | **Cured via CTx** |  |  |  |  |  |  |  |  |  |  | remainder | ctx_cure_dur | b_mort_c |
|  | **Relapsed/**  **Reinfected** |  |  |  |  |  |  |  |  |  |  | ctx_cure_rate*ctx_uptake | remainder | b_mort_c |
|  | **Dead** |  |  |  |  |  |  |  |  |  |  |  |  | 1 |

**Table A4. Calculation of the force of HIV infection per semiannual model cycle.**

| a_0_ | number of people who are uninfected in the first model cycle |
| --- | --- |
| b_0_ | number of people with early HIV, pre-ART in the first model cycle |
| c_0_ | number of people with chronic HIV, pre-ART in the first model cycle |
| d_0_ | number of people with AIDS in the first model cycle |
| e_0_ | number of people on ART in the first model cycle |
| f_0_ | number of people who have discontinued ART in the first model cycle |
|  |  |
| a_n_ | number of people who are uninfected or cured per subsequent model cycle |
| b_n_ | number of people with early HIV, pre-ART per subsequent model cycle |
| c_n_ | number of people with chronic HIV, pre-ART per subsequent model cycle |
| d_n_ | number of people with AIDS per subsequent model cycle |
| e_n_ | number of people on ART per subsequent model cycle |
| f_n_ | number of people who discontinued ART have per subsequent model cycle |
|  |  |
|  |  |
| inc_00 (semiannual HIV incidence rate per person) | semiannual HIV incidence rate per person = inc_0/1000/2 |
|  |  |
| rt_HIV (imputed risk of HIV transmission) | inc_00/SUMPRODUCT((a_0_,b_0_,c_0_,d_0_,e_0_,f_0_)/SUM(a_0_,b_0_,c_0_,d_0_,e_0_,f_0_),(0, transm_noart_early, transm_noart_chronic, transm_noart_aids, trans_art_chronic, transm_noart_disc) |
|  |  |
| inc_c (HIV incidence per semiannual model cycle) | SUMPRODUCT((a_n_,b_n_,c_n_,d_n_,e_n_,f_n_)/SUM(a_n_,b_n_,c_n_,d_n_,e_n_,f_n_),(0, transm_noart_early, transm_noart_chronic, transm_noart_aids, trans_art_chronic, transm_noart_disc) * rt_HIV |
|  |  |
| force of infection coefficient | MAX(0,1-(cov_oprep*((1+rate_oprep)^t)*(1-eff_oprep))*(cov_laprep*((1+rate_laprep)^t)*(1-eff_laprep))*(cov_cond*((1+rate_cond)^t)*(1-eff_cond))*(cov_vacc*(1-eff_vacc))) |

**Figure A2. Model Input: Number of 15-Year-Olds Entering the Open Cohort Model per Year, by Country^39^**

**Figure A3. Model Input: country-specific, age-weighted background mortality per model cycle over an 80-year time horizon.^40,41^**

**Figure A4. Model Input: country-specific average population age per model cycle over an 80-year time horizon.^39^**

**Figure A5. Model Predicted HIV Prevalence, ART Coverage, and AIDS Deaths, Pre- and Post-Introduction of Curative Therapy Cure^42^**

| 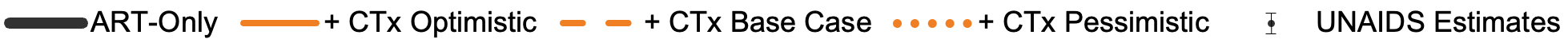 | | | | |
| --- | --- | --- | --- | --- |
|  |  | Pessimistic ART | Base Case ART | Optimistic ART |
| Ghana | HIV Prevalence (%) |  |  |  |
|  | ART Coverage (%) |  |  |  |
|  | AIDS Deaths (#) |  |  |  |
| India | HIV Prevalence (%) |  |  |  |
|  | ART Coverage (%) |  |  |  |
|  | AIDS Deaths (#) |  |  |  |
| Kenya | HIV Prevalence (%) |  |  |  |
|  | ART Coverage (%) |  |  |  |
|  | AIDS Deaths (#) |  |  |  |
| Nigeria | HIV Prevalence (%) |  |  |  |
|  | ART Coverage (%) |  |  |  |
|  | AIDS Deaths (#) |  |  |  |
| South Africa | HIV Prevalence (%) |  |  |  |
|  | ART Coverage (%) |  |  |  |
|  | AIDS Deaths (#) |  |  |  |
| Uganda | HIV Prevalence (%) |  |  |  |
|  | ART Coverage (%) |  |  |  |
|  | AIDS Deaths (#) |  |  |  |
| Zambia | HIV Prevalence (%) |  |  |  |
|  | ART Coverage (%) |  |  |  |
|  | AIDS Deaths (#) |  |  |  |
| France | HIV Prevalence (%) |  |  |  |
|  | ART Coverage (%) |  |  |  |
|  | AIDS Deaths (#) |  |  |  |
| Germany | HIV Prevalence (%) |  |  |  |
|  | ART Coverage (%) |  |  |  |
|  | AIDS Deaths (#) |  |  |  |
| Italy | HIV Prevalence (%) |  |  |  |
|  | ART Coverage (%) |  |  |  |
|  | AIDS Deaths (#) |  |  |  |
| Spain | HIV Prevalence (%) |  |  |  |
|  | ART Coverage (%) |  |  |  |
|  | AIDS Deaths (#) |  |  |  |
| United States | HIV Prevalence (%) |  |  |  |
|  | ART Coverage (%) |  |  |  |
|  | AIDS Deaths (#) |  |  |  |

ART coverage refers to the percentage of adults and children living with HIV currently receiving antiretroviral combination therapy in accordance with the nationally approved treatment protocols (or WHO/UNAIDS standards), ARV regimens prescribed for post exposure prophylaxis are excluded, among the total estimated number of adults and children living with HIV.

**Table A5. Model Results for Ghana**

| Outcomes: CTx+ART vs. ART-Only: Ghana | Calculation | Pessimistic CTx,  Pessimistic ART | Pessimistic CTx,  Base Case ART | Pessimistic CTx, Optimistic ART | Base Case CTx,  Pessimistic ART | Base Case CTx,  Base Case ART | Base Case CTx, Optimistic ART | Optimistic CTx,  Pessimistic ART | Optimistic CTx,  Base Case ART | Optimistic CTx, Optimistic ART |
| --- | --- | --- | --- | --- | --- | --- | --- | --- | --- | --- |
| Administered CTx Doses, 2030-2040 |  | 182,648 | 340,990 | 359,411 | 288,127 | 362,367 | 408,386 | 423,574 | 409,243 | 398,297 |
| Total Infections Prevented | A | 5,219,586 (-42%) | 1,904,722 (-47%) | -98,388 (+13%) | 9,154,067 (-74%) | 3,125,245 (-77%) | 243,873 (-31%) | 12,014,993 (-97%) | 3,722,308 (-91%) | 428,822 (-55%) |
| Infections Prevented/1000 Person-Years |  | 1.93 | 0.68 | -0.03 | 3.36 | 1.11 | 0.09 | 4.38 | 1.33 | 0.15 |
| Life Years Gained/1000* |  | 5,289 (+0.88%) | 1,347 (+0.22%) | 56 (+0.01%) | 11,118 (+1.44%) | 2,765 (+0.35%) | 226 (+0.03%) | 17,601 (+2.13%) | 5,207 (+0.62%) | 1,311 (+0.16%) |
| Total Deaths Averted | B | 2,455,464 (-42%) | 557,886 (-42%) | 4,062 (-2%) | 4,264,249 (-73%) | 905,297 (-68%) | 30,155 (-11%) | 5,667,600 (-97%) | 1,169,421 (-88%) | 106,944 (-40%) |
| Deaths Averted/Infection Prevented | B/A | 0.47 | 0.29 | -0.04 | 0.47 | 0.29 | 0.12 | 0.47 | 0.31 | 0.25 |
| DALYs Averted/1000* | C | 7,909 (-1.29%) | 1,577 (-0.26%) | -629 (+0.10%) | 17,633 (-2.22%) | 5,198 (-0.66%) | 888 (-0.11%) | 27,074 (-3.17%) | 8,677 (-1.02%) | 2,468 (-0.29%) |
| DALYs Averted/Infection Prevented* | C/A | 1.5 | 0.8 | 6.4 | 1.9 | 1.7 | 3.6 | 2.3 | 2.3 | 5.8 |
| Incremental HIV^†^ Cost/1000 | D | -$8.6 M (-27.0%) | -$3.8 M (-18.0%) | $0.2 M (+0.3%) | -$19.9 M (-54.9%) | -$9.7 M (-37.6%) | -$2.8 M (-4.1%) | -$28.9 M (-77.0%) | -$13.5 M (-49.3%) | -$4.5 M (-6.6%) |
| Incremental ART Cost/1000* | E | $7 M (+298%) | $ M (-13%) | $ M (-7%) | -$1 M (-52%) | -$2 M (-83%) | $ M (-67%) | -$3 M (-98%) | -$2 M (-97%) | -$1 M (-91%) |
| *Value-Based Price/Patient: Curative Therapy* |  | $24,950 | $12,504 | $0** | $32,513 | $12,166 | $3,241 | $117,415 | $53,309 | $17,104 |
| Incremental CTx Cost/1000 (using VBP)* | F | $6 M | $5 M | -$1 M | $31 M | $14 M | $4 M | $47 M | $21 M | $6 M |
| Incremental HIV+ART+CTx Cost/1000* | D+E+F | $4 M (+12.8%) | $1 M (+3.8%) | $ M (-0.6%) | $10 M (+24.9%) | $3 M (+10.2%) | $ M (+0.7%) | $15 M (+36.9%) | $5 M (+16.1%) | $1 M (+2.0%) |
| Total Cost per DALY Averted | (D+E+F)/C | $552 | $552 | $552 | $552 | $552 | $552 | $552 | $552 | $552 |

**Table A6. Model Results for India**

| Outcomes: CTx+ART vs. ART-Only: India | Calculation | Pessimistic CTx,  Pessimistic ART | Pessimistic CTx,  Base Case ART | Pessimistic CTx, Optimistic ART | Base Case CTx,  Pessimistic ART | Base Case CTx,  Base Case ART | Base Case CTx, Optimistic ART | Optimistic CTx,  Pessimistic ART | Optimistic CTx,  Base Case ART | Optimistic CTx, Optimistic ART |
| --- | --- | --- | --- | --- | --- | --- | --- | --- | --- | --- |
| Administered CTx Doses, 2030-2040 |  | 461,455 | 1,120,898 | 1,246,119 | 1,198,120 | 1,632,180 | 1,819,515 | 1,779,327 | 1,743,870 | 1,702,974 |
| Total Infections Prevented | A | 1,855,084 (-29%) | 1,147,905 (-34%) | -87,116 (+7%) | 4,066,570 (-64%) | 2,026,286 (-59%) | 248,907 (-20%) | 5,415,640 (-85%) | 2,500,102 (-73%) | 411,617 (-33%) |
| Infections Prevented/1000 Person-Years |  | 0.02 | 0.01 | 0.00 | 0.05 | 0.02 | 0.00 | 0.07 | 0.03 | 0.01 |
| Life Years Gained/1000* |  | 4,381 (+0.03%) | 2,048 (+0.01%) | 188 (+0.00%) | 12,825 (+0.05%) | 4,453 (+0.02%) | 447 (+0.00%) | 23,108 (+0.09%) | 8,837 (+0.03%) | 2,266 (+0.01%) |
| Total Deaths Averted | B | 1,397,729 (-28%) | 582,698 (-26%) | 17,361 (-2%) | 2,952,452 (-59%) | 923,868 (-42%) | 49,240 (-5%) | 4,090,974 (-81%) | 1,274,275 (-58%) | 172,002 (-16%) |
| Deaths Averted/Infection Prevented | B/A | 0.75 | 0.51 | -0.20 | 0.73 | 0.46 | 0.20 | 0.76 | 0.51 | 0.42 |
| DALYs Averted/1000* | C | 5,627 (-0.03%) | 1,779 (-0.01%) | -1,256 (+0.01%) | 19,480 (-0.08%) | 8,513 (-0.03%) | 2,364 (-0.01%) | 34,182 (-0.13%) | 15,084 (-0.06%) | 5,414 (-0.02%) |
| DALYs Averted/Infection Prevented* | C/A | 3.0 | 1.5 | 14.4 | 4.8 | 4.2 | 9.5 | 6.3 | 6.0 | 13.2 |
| Incremental HIV^†^ Cost/1000 | D | -$1.9 M (-5.8%) | -$1.8 M (-2.4%) | $0.0 M (0.0%) | -$10.0 M (-18.1%) | -$9.2 M (-8.4%) | -$5.9 M (-1.2%) | -$16.4 M (-25.5%) | -$13.3 M (-11.0%) | -$8.7 M (-1.6%) |
| Incremental ART Cost/1000* | E | $38 M (+394%) | $1 M (+5%) | $0 M (-8%) | -$3 M (-18%) | -$13 M (-72%) | -$4 M (-62%) | -$16 M (-93%) | -$18 M (-94%) | -$8 M (-88%) |
| *Value-Based Price/Patient: Curative Therapy* |  | $0** | $2,202 | $0** | $6,406 | $6,378 | $2,787 | $27,289 | $22,713 | $11,515 |
| Incremental CTx Cost/1000 (using VBP)* | F | -$33 M | $2 M | -$0 M | $20 M | $25 M | $11 M | $46 M | $37 M | $19 M |
| Incremental HIV+ART+CTx Cost/1000* | D+E+F | $2 M (+5.4%) | $1 M (+0.8%) | $0 M (-0.1%) | $8 M (+11.0%) | $3 M (+2.7%) | $1 M (+0.2%) | $14 M (+16.7%) | $6 M (+4.3%) | $2 M (+0.4%) |
| Total Cost per DALY Averted | (D+E+F)/C | $397 | $397 | $397 | $397 | $397 | $397 | $397 | $397 | $397 |

**Table A7. Model Results for Kenya**

| Outcomes: CTx+ART vs. ART-Only: Kenya | Calculation | Pessimistic CTx,  Pessimistic ART | Pessimistic CTx,  Base Case ART | Pessimistic CTx, Optimistic ART | Base Case CTx,  Pessimistic ART | Base Case CTx,  Base Case ART | Base Case CTx, Optimistic ART | Optimistic CTx,  Pessimistic ART | Optimistic CTx,  Base Case ART | Optimistic CTx, Optimistic ART |
| --- | --- | --- | --- | --- | --- | --- | --- | --- | --- | --- |
| Administered CTx Doses, 2030-2040 |  | 1,099,816 | 1,324,557 | 1,269,152 | 1,641,124 | 1,727,590 | 1,719,470 | 1,574,330 | 1,467,365 | 1,432,103 |
| Total Infections Prevented | A | 19,292,704 (-50%) | 134,013 (-4%) | -254,253 (+16%) | 33,290,571 (-86%) | 1,892,191 (-62%) | 653,994 (-41%) | 37,693,358 (-98%) | 2,309,056 (-76%) | 880,796 (-56%) |
| Infections Prevented/1000 Person-Years |  | 4.70 | 0.03 | -0.06 | 7.94 | 0.42 | 0.15 | 8.93 | 0.51 | 0.20 |
| Life Years Gained/1000* |  | 23,458 (+2.50%) | 468 (+0.05%) | 212 (+0.02%) | 44,858 (+3.59%) | 1,737 (+0.13%) | 498 (+0.04%) | 54,531 (+4.05%) | 3,041 (+0.22%) | 1,070 (+0.08%) |
| Total Deaths Averted | B | 10,028,128 (-55%) | 84,856 (-11%) | 20,871 (-5%) | 16,157,954 (-88%) | 360,237 (-45%) | 59,770 (-14%) | 18,030,978 (-98%) | 468,819 (-59%) | 97,631 (-23%) |
| Deaths Averted/Infection Prevented | B/A | 0.52 | 0.63 | -0.08 | 0.49 | 0.19 | 0.09 | 0.48 | 0.20 | 0.11 |
| DALYs Averted/1000* | C | 34,176 (-3.45%) | -1,518 (+0.15%) | -1,839 (+0.18%) | 71,749 (-5.41%) | 4,860 (-0.37%) | 2,681 (-0.21%) | 86,503 (-6.03%) | 7,312 (-0.52%) | 4,112 (-0.29%) |
| DALYs Averted/Infection Prevented* | C/A | 1.8 | -11.3 | 7.2 | 2.2 | 2.6 | 4.1 | 2.3 | 3.2 | 4.7 |
| Incremental HIV^†^ Cost/1000 | D | -$32.4 M (-31.4%) | -$0.6 M (-2.0%) | $0.6 M (+1.2%) | -$76.4 M (-65.6%) | -$12.0 M (-29.6%) | -$7.9 M (-11.9%) | -$94.8 M (-78.9%) | -$16.8 M (-37.8%) | -$11.5 M (-16.3%) |
| Incremental ART Cost/1000* | E | $50 M (+254%) | -$1 M (-8%) | $0 M (-7%) | -$15 M (-65%) | -$8 M (-79%) | -$2 M (-64%) | -$24 M (-97%) | -$10 M (-95%) | -$3 M (-87%) |
| *Value-Based Price/Patient: Curative Therapy* |  | $0** | $363 | $0** | $26,448 | $5,417 | $2,808 | $107,939 | $22,113 | $12,250 |
| Incremental CTx Cost/1000 (using VBP)* | F | -$1 M | $0 M | -$1 M | $126 M | $22 M | $11 M | $160 M | $31 M | $17 M |
| Incremental HIV+ART+CTx Cost/1000* | D+E+F | $17 M (+13.4%) | -$1 M (-2.0%) | -$1 M (-1.6%) | $35 M (+24.8%) | $2 M (+4.6%) | $1 M (+1.9%) | $42 M (+28.9%) | $4 M (+6.4%) | $2 M (+2.7%) |
| Total Cost per DALY Averted | (D+E+F)/C | $483 | $483 | $483 | $483 | $483 | $483 | $483 | $483 | $483 |

**Table A8. Model Results for Nigeria**

| Outcomes: CTx+ART vs. ART-Only: Nigeria | Calculation | Pessimistic CTx,  Pessimistic ART | Pessimistic CTx,  Base Case ART | Pessimistic CTx, Optimistic ART | Base Case CTx,  Pessimistic ART | Base Case CTx,  Base Case ART | Base Case CTx, Optimistic ART | Optimistic CTx,  Pessimistic ART | Optimistic CTx,  Base Case ART | Optimistic CTx, Optimistic ART |
| --- | --- | --- | --- | --- | --- | --- | --- | --- | --- | --- |
| Administered CTx Doses, 2030-2040 |  | 1,347,864 | 2,209,661 | 2,039,038 | 1,967,511 | 2,348,736 | 2,466,820 | 2,281,944 | 2,197,962 | 2,134,032 |
| Total Infections Prevented | A | 51,702,416 (-55%) | 12,095,995 (-55%) | -661,045 (+17%) | 81,000,192 (-86%) | 18,668,566 (-84%) | 1,541,145 (-41%) | 92,490,805 (-98%) | 20,360,112 (-92%) | 2,123,506 (-56%) |
| Infections Prevented/1000 Person-Years |  | 1.98 | 0.45 | -0.02 | 3.07 | 0.69 | 0.06 | 3.50 | 0.75 | 0.08 |
| Life Years Gained/1000* |  | 52,175 (+0.93%) | 8,422 (+0.15%) | 276 (+0.00%) | 96,658 (+1.40%) | 16,079 (+0.23%) | 1,323 (+0.02%) | 125,500 (+1.72%) | 23,991 (+0.32%) | 4,508 (+0.06%) |
| Total Deaths Averted | B | 24,492,368 (-55%) | 3,197,858 (-52%) | 20,324 (-2%) | 38,005,991 (-85%) | 4,759,811 (-77%) | 191,652 (-17%) | 43,687,044 (-98%) | 5,413,898 (-88%) | 392,128 (-35%) |
| Deaths Averted/Infection Prevented | B/A | 0.47 | 0.26 | -0.03 | 0.47 | 0.25 | 0.12 | 0.47 | 0.27 | 0.18 |
| DALYs Averted/1000* | C | 77,121 (-1.34%) | 9,376 (-0.16%) | -4,139 (+0.07%) | 150,882 (-2.13%) | 31,033 (-0.44%) | 5,546 (-0.08%) | 191,978 (-2.56%) | 42,393 (-0.57%) | 10,494 (-0.14%) |
| DALYs Averted/Infection Prevented* | C/A | 1.5 | 0.8 | 6.3 | 1.9 | 1.7 | 3.6 | 2.1 | 2.1 | 4.9 |
| Incremental HIV^†^ Cost/1000 | D | -$30.1 M (-33.6%) | -$9.9 M (-11.5%) | -$0.1 M (0.0%) | -$65.8 M (-63.7%) | -$28.3 M (-27.1%) | -$10.1 M (-1.6%) | -$82.5 M (-76.4%) | -$34.6 M (-31.5%) | -$13.5 M (-2.1%) |
| Incremental ART Cost/1000* | E | $75 M (+317%) | -$2 M (-17%) | $0 M (-7%) | -$16 M (-60%) | -$14 M (-85%) | -$2 M (-67%) | -$27 M (-98%) | -$17 M (-97%) | -$4 M (-90%) |
| *Value-Based Price/Patient: Curative Therapy* |  | $0** | $5,899 | $0** | $16,583 | $6,808 | $2,092 | $69,733 | $28,925 | $9,542 |
| Incremental CTx Cost/1000 (using VBP)* | F | -$28 M | $14 M | -$1 M | $115 M | $49 M | $14 M | $152 M | $61 M | $19 M |
| Incremental HIV+ART+CTx Cost/1000* | D+E+F | $17 M (+15.2%) | $2 M (+2.1%) | -$1 M (-0.2%) | $34 M (+25.9%) | $7 M (+5.7%) | $1 M (+0.2%) | $43 M (+31.6%) | $9 M (+7.4%) | $2 M (+0.4%) |
| Total Cost per DALY Averted | (D+E+F)/C | $223 | $223 | $223 | $223 | $223 | $223 | $223 | $223 | $223 |

**Table A9. Model Results for South Africa**

| Outcomes: CTx+ART vs. ART-Only: South Africa | Calculation | Pessimistic CTx,  Pessimistic ART | Pessimistic CTx,  Base Case ART | Pessimistic CTx, Optimistic ART | Base Case CTx,  Pessimistic ART | Base Case CTx,  Base Case ART | Base Case CTx, Optimistic ART | Optimistic CTx,  Pessimistic ART | Optimistic CTx,  Base Case ART | Optimistic CTx, Optimistic ART |
| --- | --- | --- | --- | --- | --- | --- | --- | --- | --- | --- |
| Administered CTx Doses, 2030-2040 |  | 4,521,314 | 5,595,081 | 5,387,416 | 7,145,044 | 7,506,204 | 7,522,257 | 7,200,875 | 6,593,506 | 6,443,313 |
| Total Infections Prevented | A | 14,872,894 (-23%) | -144,477 (+1%) | -1,215,392 (+13%) | 33,340,279 (-53%) | 8,783,006 (-54%) | 3,287,400 (-36%) | 57,850,634 (-91%) | 11,380,510 (-70%) | 4,594,543 (-50%) |
| Infections Prevented/1000 Person-Years |  | 6.42 | -0.03 | -0.35 | 13.06 | 2.58 | 0.95 | 20.60 | 3.33 | 1.33 |
| Life Years Gained/1000* |  | 42,834 (+7.34%) | 1,695 (+0.24%) | 1,006 (+0.14%) | 96,232 (+11.07%) | 7,286 (+0.73%) | 2,282 (+0.23%) | 144,577 (+14.95%) | 13,767 (+1.25%) | 5,195 (+0.47%) |
| Total Deaths Averted | B | 13,100,643 (-37%) | 219,080 (-6%) | 80,862 (-4%) | 23,151,705 (-66%) | 1,533,120 (-39%) | 255,349 (-11%) | 33,257,944 (-95%) | 2,141,208 (-55%) | 461,820 (-21%) |
| Deaths Averted/Infection Prevented | B/A | 0.88 | -1.52 | -0.07 | 0.69 | 0.17 | 0.08 | 0.57 | 0.19 | 0.10 |
| DALYs Averted/1000* | C | 55,611 (-8.02%) | -4,000 (+0.55%) | -4,788 (+0.66%) | 148,987 (-14.27%) | 20,192 (-1.97%) | 11,340 (-1.11%) | 223,637 (-18.76%) | 32,146 (-2.84%) | 18,206 (-1.62%) |
| DALYs Averted/Infection Prevented* | C/A | 3.7 | 27.7 | 3.9 | 4.5 | 2.3 | 3.4 | 3.9 | 2.8 | 4.0 |
| Incremental HIV^†^ Cost/1000 | D | -$55.5 M (-10.4%) | -$6.2 M (-2.6%) | $2.9 M (+1.4%) | -$335.5 M (-50.7%) | -$170.9 M (-50.8%) | -$131.6 M (-43.3%) | -$558.2 M (-80.1%) | -$240.1 M (-64.8%) | -$187.3 M (-55.6%) |
| Incremental ART Cost/1000* | E | $141 M (+306%) | -$2 M (-7%) | $0 M (-6%) | -$29 M (-50%) | -$27 M (-78%) | -$6 M (-63%) | -$60 M (-96%) | -$36 M (-94%) | -$11 M (-87%) |
| *Value-Based Price/Patient: Curative Therapy* |  | $19,977 | $0** | $0** | $40,867 | $14,961 | $10,460 | $197,611 | $60,939 | $42,208 |
| Incremental CTx Cost/1000 (using VBP)* | F | $94 M | -$5 M | -$18 M | $846 M | $263 M | $175 M | $1,340 M | $380 M | $257 M |
| Incremental HIV+ART+CTx Cost/1000* | D+E+F | $180 M (+31.0%) | -$13 M (-4.9%) | -$15 M (-7.2%) | $481 M (+66.8%) | $65 M (+17.6%) | $37 M (+11.7%) | $722 M (+95.1%) | $104 M (+25.4%) | $59 M (+16.8%) |
| Total Cost per DALY Averted | (D+E+F)/C | $3,228 | $3,228 | $3,228 | $3,228 | $3,228 | $3,228 | $3,228 | $3,228 | $3,228 |

**Table A10. Model Results for Uganda**

| Outcomes: CTx+ART vs. ART-Only: Uganda | Calculation | Pessimistic CTx,  Pessimistic ART | Pessimistic CTx,  Base Case ART | Pessimistic CTx, Optimistic ART | Base Case CTx,  Pessimistic ART | Base Case CTx,  Base Case ART | Base Case CTx, Optimistic ART | Optimistic CTx,  Pessimistic ART | Optimistic CTx,  Base Case ART | Optimistic CTx, Optimistic ART |
| --- | --- | --- | --- | --- | --- | --- | --- | --- | --- | --- |
| Administered CTx Doses, 2030-2040 |  | 1,944,600 | 2,008,229 | 1,597,735 | 2,024,031 | 2,046,076 | 1,907,951 | 1,793,698 | 1,623,762 | 1,517,733 |
| Total Infections Prevented | A | 34,732,539 (-33%) | 4,928,570 (-32%) | -768,930 (+24%) | 69,180,916 (-65%) | 12,939,004 (-84%) | 1,687,054 (-53%) | 104,814,289 (-99%) | 14,158,266 (-92%) | 2,128,382 (-67%) |
| Infections Prevented/1000 Person-Years |  | 8.27 | 0.87 | -0.13 | 15.25 | 2.28 | 0.30 | 21.69 | 2.49 | 0.37 |
| Life Years Gained/1000* |  | 65,508 (+6.01%) | 2,681 (+0.22%) | 264 (+0.02%) | 123,740 (+9.17%) | 6,700 (+0.45%) | 814 (+0.05%) | 159,452 (+11.20%) | 9,304 (+0.59%) | 1,623 (+0.10%) |
| Total Deaths Averted | B | 24,421,402 (-45%) | 893,777 (-37%) | 24,962 (-5%) | 41,043,261 (-76%) | 1,895,034 (-78%) | 118,824 (-25%) | 53,801,095 (-99%) | 2,117,798 (-87%) | 170,740 (-35%) |
| Deaths Averted/Infection Prevented | B/A | 0.70 | 0.18 | -0.03 | 0.59 | 0.15 | 0.07 | 0.51 | 0.15 | 0.08 |
| DALYs Averted/1000* | C | 89,331 (-7.22%) | -250 (+0.02%) | -3,404 (+0.27%) | 189,250 (-12.14%) | 15,943 (-1.05%) | 4,128 (-0.27%) | 245,257 (-14.69%) | 20,322 (-1.27%) | 5,953 (-0.37%) |
| DALYs Averted/Infection Prevented* | C/A | 2.6 | -0.1 | 4.4 | 2.7 | 1.2 | 2.4 | 2.3 | 1.4 | 2.8 |
| Incremental HIV^†^ Cost/1000 | D | -$37.5 M (-25.0%) | -$6.3 M (-14.5%) | $0.7 M (+0.7%) | -$104.0 M (-65.2%) | -$26.3 M (-51.4%) | -$8.6 M (-8.3%) | -$146.5 M (-90.4%) | -$32.2 M (-60.3%) | -$11.8 M (-11.0%) |
| Incremental ART Cost/1000* | E | $46 M (+311%) | -$1 M (-18%) | $0 M (-6%) | -$10 M (-66%) | -$5 M (-87%) | -$1 M (-67%) | -$16 M (-99%) | -$5 M (-97%) | -$1 M (-89%) |
| *Value-Based Price/Patient: Curative Therapy* |  | $629 | $2,828 | $0** | $15,016 | $5,616 | $2,023 | $113,594 | $26,065 | $9,353 |
| Incremental CTx Cost/1000 (using VBP)* | F | $2 M | $7 M | -$1 M | $137 M | $33 M | $10 M | $192 M | $40 M | $13 M |
| Incremental HIV+ART+CTx Cost/1000* | D+E+F | $11 M (+6.5%) | $ M (-0.1%) | $ M (-0.4%) | $23 M (+12.9%) | $2 M (+3.4%) | $ M (+0.5%) | $29 M (+16.5%) | $2 M (+4.1%) | $1 M (+0.7%) |
| Total Cost per DALY Averted | (D+E+F)/C | $120 | $120 | $120 | $120 | $120 | $120 | $120 | $120 | $120 |

**Table A11. Model Results for Zambia**

| Outcomes: CTx+ART vs. ART-Only: Zambia | Calculation | Pessimistic CTx,  Pessimistic ART | Pessimistic CTx,  Base Case ART | Pessimistic CTx, Optimistic ART | Base Case CTx,  Pessimistic ART | Base Case CTx,  Base Case ART | Base Case CTx, Optimistic ART | Optimistic CTx,  Pessimistic ART | Optimistic CTx,  Base Case ART | Optimistic CTx, Optimistic ART |
| --- | --- | --- | --- | --- | --- | --- | --- | --- | --- | --- |
| Administered CTx Doses, 2030-2040 |  | 1,085,387 | 1,364,993 | 1,196,402 | 1,451,154 | 1,567,388 | 1,526,672 | 1,446,857 | 1,346,539 | 1,282,162 |
| Total Infections Prevented | A | 12,632,159 (-34%) | 1,907,435 (-25%) | -491,608 (+20%) | 24,948,960 (-68%) | 5,705,646 (-76%) | 1,142,154 (-48%) | 35,494,277 (-97%) | 6,501,124 (-86%) | 1,471,710 (-61%) |
| Infections Prevented/1000 Person-Years |  | 8.20 | 0.95 | -0.24 | 15.09 | 2.80 | 0.55 | 20.40 | 3.19 | 0.71 |
| Life Years Gained/1000* |  | 23,381 (+6.02%) | 1,466 (+0.33%) | 186 (+0.04%) | 46,269 (+9.37%) | 4,023 (+0.73%) | 700 (+0.13%) | 61,986 (+11.80%) | 6,242 (+1.07%) | 1,533 (+0.26%) |
| Total Deaths Averted | B | 8,520,795 (-45%) | 442,716 (-28%) | 8,534 (-2%) | 14,432,917 (-76%) | 1,051,004 (-66%) | 103,672 (-21%) | 18,560,491 (-98%) | 1,249,567 (-79%) | 162,001 (-33%) |
| Deaths Averted/Infection Prevented | B/A | 0.67 | 0.23 | -0.02 | 0.58 | 0.18 | 0.09 | 0.52 | 0.19 | 0.11 |
| DALYs Averted/1000* | C | 31,739 (-7.17%) | -117 (+0.03%) | -2,067 (+0.46%) | 71,133 (-12.41%) | 9,262 (-1.65%) | 3,127 (-0.56%) | 95,020 (-15.30%) | 12,885 (-2.17%) | 4,815 (-0.81%) |
| DALYs Averted/Infection Prevented* | C/A | 2.5 | -0.1 | 4.2 | 2.9 | 1.6 | 2.7 | 2.7 | 2.0 | 3.3 |
| Incremental HIV^†^ Cost/1000 | D | -$15.8 M (-22.0%) | -$3.3 M (-10.6%) | $0.3 M (+0.4%) | -$48.5 M (-60.0%) | -$17.6 M (-45.1%) | -$7.7 M (-9.0%) | -$69.1 M (-83.0%) | -$22.5 M (-54.3%) | -$10.6 M (-12.0%) |
| Incremental ART Cost/1000* | E | $82 M (+317%) | -$1 M (-13%) | $ M (-7%) | -$17 M (-59%) | -$12 M (-83%) | -$2 M (-66%) | -$30 M (-98%) | -$14 M (-96%) | -$3 M (-88%) |
| *Value-Based Price/Patient: Curative Therapy* |  | $0** | $3,345 | $0** | $21,186 | $8,276 | $3,109 | $112,140 | $34,787 | $13,767 |
| Incremental CTx Cost/1000 (using VBP)* | F | -$47 M | $5 M | -$1 M | $108 M | $35 M | $12 M | $155 M | $45 M | $17 M |
| Incremental HIV+ART+CTx Cost/1000* | D+E+F | $19 M (+19.2%) | $ M (-0.2%) | -$1 M (-1.6%) | $42 M (+38.2%) | $5 M (+10.3%) | $2 M (+2.1%) | $56 M (+49.4%) | $8 M (+13.5%) | $3 M (+3.1%) |
| Total Cost per DALY Averted | (D+E+F)/C | $591 | $591 | $591 | $591 | $591 | $591 | $591 | $591 | $591 |

**Table A12. Model Results for France**

| Outcomes: CTx+ART vs. ART-Only: France | Calculation | Pessimistic CTx,  Pessimistic ART | Pessimistic CTx,  Base Case ART | Pessimistic CTx, Optimistic ART | Base Case CTx,  Pessimistic ART | Base Case CTx,  Base Case ART | Base Case CTx, Optimistic ART | Optimistic CTx,  Pessimistic ART | Optimistic CTx,  Base Case ART | Optimistic CTx, Optimistic ART |
| --- | --- | --- | --- | --- | --- | --- | --- | --- | --- | --- |
| Administered CTx Doses, 2030-2040 |  | 57,225 | 145,077 | 133,792 | 99,852 | 158,847 | 163,313 | 149,565 | 137,652 | 131,344 |
| Total Infections Prevented | A | 2,845,381 (-41%) | 868,301 (-63%) | -22,644 (+11%) | 5,316,687 (-76%) | 1,186,171 (-86%) | 83,244 (-39%) | 6,855,415 (-98%) | 1,275,518 (-92%) | 116,282 (-54%) |
| Infections Prevented/1000 Person-Years |  | 0.66 | 0.20 | -0.01 | 1.23 | 0.27 | 0.02 | 1.58 | 0.29 | 0.03 |
| Life Years Gained/1000* |  | 3,319 (+0.38%) | 765 (+0.09%) | 31 (+0.00%) | 7,681 (+0.66%) | 1,240 (+0.11%) | 103 (+0.01%) | 11,617 (+0.93%) | 1,683 (+0.13%) | 248 (+0.02%) |
| Total Deaths Averted | B | 1,451,889 (-41%) | 264,054 (-67%) | 4,532 (-8%) | 2,690,116 (-77%) | 325,564 (-83%) | 10,869 (-19%) | 3,468,414 (-99%) | 353,067 (-90%) | 18,385 (-33%) |
| Deaths Averted/Infection Prevented | B/A | 0.51 | 0.30 | -0.20 | 0.51 | 0.27 | 0.13 | 0.51 | 0.28 | 0.16 |
| DALYs Averted/1000* | C | 4,841 (-0.54%) | 1,022 (-0.11%) | -104 (+0.01%) | 11,447 (-0.98%) | 2,174 (-0.19%) | 376 (-0.03%) | 16,920 (-1.34%) | 2,810 (-0.22%) | 624 (-0.05%) |
| DALYs Averted/Infection Prevented* | C/A | 1.7 | 1.2 | 4.6 | 2.2 | 1.8 | 4.5 | 2.5 | 2.2 | 5.4 |
| Incremental HIV^†^ Cost/1000 | D | -$75.0 M (-23.3%) | -$30.9 M (-14.6%) | $0.5 M (+0.0%) | -$193.4 M (-53.1%) | -$71.4 M (-27.2%) | -$20.5 M (-1.7%) | -$288.9 M (-76.3%) | -$88.7 M (-31.7%) | -$29.3 M (-2.4%) |
| Incremental ART Cost/1000* | E | $425 M (+637%) | -$10 M (-15%) | -$1 M (-7%) | -$7 M (-10%) | -$64 M (-85%) | -$10 M (-66%) | -$76 M (-98%) | -$77 M (-97%) | -$16 M (-89%) |
| *Value-Based Price/Patient: Curative Therapy* |  | $0** | $657,324 | $0** | $1,547,065 | $513,293 | $118,433 | $9,217,161 | $2,467,945 | $643,335 |
| Incremental CTx Cost/1000 (using VBP)* | F | -$84 M | $97 M | -$6 M | $831 M | $255 M | $51 M | $1,296 M | $321 M | $80 M |
| Incremental HIV+ART+CTx Cost/1000* | D+E+F | $266 M (+68.6%) | $56 M (+20.4%) | -$6 M (-0.5%) | $630 M (+143.2%) | $120 M (+35.3%) | $21 M (+1.7%) | $931 M (+203.7%) | $155 M (+43.1%) | $34 M (+2.8%) |
| Total Cost per DALY Averted | (D+E+F)/C | $55,027 | $55,027 | $55,027 | $55,027 | $55,027 | $55,027 | $55,027 | $55,027 | $55,027 |

**Table A13. Model Results for Germany**

| Outcomes: CTx+ART vs. ART-Only: Germany | Calculation | Pessimistic CTx,  Pessimistic ART | Pessimistic CTx,  Base Case ART | Pessimistic CTx, Optimistic ART | Base Case CTx,  Pessimistic ART | Base Case CTx,  Base Case ART | Base Case CTx, Optimistic ART | Optimistic CTx,  Pessimistic ART | Optimistic CTx,  Base Case ART | Optimistic CTx, Optimistic ART |
| --- | --- | --- | --- | --- | --- | --- | --- | --- | --- | --- |
| Administered CTx Doses, 2030-2040 |  | 12,025 | 29,124 | 27,169 | 28,692 | 47,412 | 49,302 | 56,297 | 51,993 | 49,629 |
| Total Infections Prevented | A | 118,019 (-23%) | 39,620 (-26%) | 205 (0%) | 284,430 (-55%) | 76,059 (-50%) | 11,922 (-17%) | 460,048 (-89%) | 103,056 (-68%) | 22,276 (-33%) |
| Infections Prevented/1000 Person-Years |  | 0.05 | 0.02 | 0.00 | 0.13 | 0.04 | 0.01 | 0.21 | 0.05 | 0.01 |
| Life Years Gained/1000* |  | 109 (+0.03%) | 38 (+0.01%) | 7 (+0.00%) | 334 (+0.05%) | 85 (+0.01%) | 20 (+0.00%) | 690 (+0.10%) | 158 (+0.02%) | 49 (+0.01%) |
| Total Deaths Averted | B | 52,520 (-25%) | 12,722 (-27%) | 672 (-3%) | 120,682 (-58%) | 21,299 (-46%) | 2,277 (-11%) | 191,403 (-91%) | 29,588 (-63%) | 5,035 (-23%) |
| Deaths Averted/Infection Prevented | B/A | 0.45 | 0.32 | 3.27 | 0.42 | 0.28 | 0.19 | 0.42 | 0.29 | 0.23 |
| DALYs Averted/1000* | C | 176 (-0.05%) | 49 (-0.01%) | -2 (+0.00%) | 591 (-0.10%) | 179 (-0.03%) | 62 (-0.01%) | 1,185 (-0.17%) | 311 (-0.04%) | 124 (-0.02%) |
| DALYs Averted/Infection Prevented* | C/A | 1.5 | 1.2 | -11.4 | 2.1 | 2.4 | 5.2 | 2.6 | 3.0 | 5.5 |
| Incremental HIV^†^ Cost/1000 | D | -$2.7 M (-11.0%) | -$1.2 M (-5.9%) | -$0.1 M (-0.1%) | -$11.2 M (-30.7%) | -$5.6 M (-16.7%) | -$2.9 M (-3.0%) | -$23.2 M (-57.0%) | -$9.4 M (-24.2%) | -$5.1 M (-5.1%) |
| Incremental ART Cost/1000* | E | $26 M (+233%) | $ M (-2%) | $ M (-6%) | -$4 M (-23%) | -$10 M (-67%) | -$3 M (-54%) | -$16 M (-93%) | -$16 M (-92%) | -$6 M (-84%) |
| *Value-Based Price/Patient: Curative Therapy* |  | $0** | $236,214 | $0** | $741,369 | $310,920 | $124,864 | $2,721,230 | $1,070,909 | $477,901 |
| Incremental CTx Cost/1000 (using VBP)* | F | -$8 M | $6 M | -$0 M | $67 M | $31 M | $11 M | $143 M | $52 M | $22 M |
| Incremental HIV+ART+CTx Cost/1000* | D+E+F | $16 M (+43.1%) | $4 M (+14.5%) | $ M (-0.3%) | $52 M (+100.5%) | $16 M (+32.4%) | $5 M (+5.4%) | $104 M (+180.6%) | $27 M (+49.2%) | $11 M (+10.0%) |
| Total Cost per DALY Averted | (D+E+F)/C | $88,043 | $88,043 | $88,043 | $88,043 | $88,043 | $88,043 | $88,043 | $88,043 | $88,043 |

**Table A14. Model Results for Italy**

| Outcomes: CTx+ART vs. ART-Only: Italy | Calculation | Pessimistic CTx,  Pessimistic ART | Pessimistic CTx,  Base Case ART | Pessimistic CTx, Optimistic ART | Base Case CTx,  Pessimistic ART | Base Case CTx,  Base Case ART | Base Case CTx, Optimistic ART | Optimistic CTx,  Pessimistic ART | Optimistic CTx,  Base Case ART | Optimistic CTx, Optimistic ART |
| --- | --- | --- | --- | --- | --- | --- | --- | --- | --- | --- |
| Administered CTx Doses, 2030-2040 |  | 32,908 | 42,463 | 40,915 | 71,825 | 78,268 | 78,143 | 85,983 | 80,163 | 78,269 |
| Total Infections Prevented | A | 275,675 (-40%) | 1,701 (-2%) | -3,373 (+5%) | 523,143 (-77%) | 29,754 (-31%) | 13,508 (-19%) | 621,133 (-91%) | 44,731 (-46%) | 22,366 (-31%) |
| Infections Prevented/1000 Person-Years |  | 0.17 | 0.001 | -0.002 | 0.33 | 0.02 | 0.01 | 0.39 | 0.03 | 0.01 |
| Life Years Gained/1000* |  | 213 (+0.080%) | 11 (+0.004%) | 8 (+0.003%) | 511 (+0.114%) | 34 (+0.008%) | 18 (+0.004%) | 717 (+0.138%) | 73 (+0.014%) | 38 (+0.007%) |
| Total Deaths Averted | B | 106,599 (-41%) | 1,089 (-3%) | 276 (-1%) | 196,694 (-76%) | 5,595 (-17%) | 1,427 (-5%) | 233,965 (-91%) | 9,857 (-30%) | 3,231 (-12%) |
| Deaths Averted/Infection Prevented | B/A | 0.39 | 0.64 | -0.08 | 0.38 | 0.19 | 0.11 | 0.38 | 0.22 | 0.14 |
| DALYs Averted/1000* | C | 372 (-0.140%) | 5 (-0.002%) | -1 (+0.000%) | 957 (-0.214%) | 108 (-0.024%) | 75 (-0.017%) | 1,325 (-0.255%) | 198 (-0.038%) | 135 (-0.026%) |
| DALYs Averted/Infection Prevented* | C/A | 1.3 | 3.0 | 0.3 | 1.8 | 3.6 | 5.6 | 2.1 | 4.4 | 6.0 |
| Incremental HIV^†^ Cost/1000 | D | -$6.5 M (-20.7%) | -$0.1 M (-0.4%) | $0.1 M (+0.1%) | -$19.2 M (-42.2%) | -$3.7 M (-10.0%) | -$2.9 M (-2.6%) | -$27.0 M (-52.8%) | -$6.3 M (-14.6%) | -$4.9 M (-4.2%) |
| Incremental ART Cost/1000* | E | $13.4 M (+123%) | -$0.3 M (-6%) | -$0.1 M (-6%) | -$9.2 M (-57%) | -$5.9 M (-66%) | -$1.9 M (-54%) | -$16.7 M (-93%) | -$9.4 M (-90%) | -$3.9 M (-82%) |
| *Value-Based Price/Patient: Curative Therapy* |  | $370,721 | $21,647 | $0** | $490,468 | $102,357 | $59,981 | $1,360,842 | $338,009 | $211,571 |
| Incremental CTx Cost/1000 (using VBP)* | F | $12 M | $1 M | -$0.1 M | $76 M | $15 M | $9 M | $110 M | $26 M | $16 M |
| Incremental HIV+ART+CTx Cost/1000* | D+E+F | $19 M (+44.0%) | $ M (+1.0%) | $ M (-0.1%) | $48 M (+77.5%) | $5 M (+11.8%) | $4 M (+3.3%) | $66 M (+95.9%) | $10 M (+18.4%) | $7 M (+5.6%) |
| Total Cost per DALY Averted | (D+E+F)/C | $50,000 | $50,000 | $50,000 | $50,000 | $50,000 | $50,000 | $50,000 | $50,000 | $50,000 |

**Table A15. Model Results for Spain**

| Outcomes: CTx+ART vs. ART-Only: Spain | Calculation | Pessimistic CTx,  Pessimistic ART | Pessimistic CTx,  Base Case ART | Pessimistic CTx, Optimistic ART | Base Case CTx,  Pessimistic ART | Base Case CTx,  Base Case ART | Base Case CTx, Optimistic ART | Optimistic CTx,  Pessimistic ART | Optimistic CTx,  Base Case ART | Optimistic CTx, Optimistic ART |
| --- | --- | --- | --- | --- | --- | --- | --- | --- | --- | --- |
| Administered CTx Doses, 2030-2040 |  | 31,597 | 66,597 | 58,980 | 66,465 | 96,512 | 97,114 | 109,268 | 98,152 | 92,652 |
| Total Infections Prevented | A | 397,166 (-25%) | 71,601 (-24%) | -4,905 (+4%) | 985,893 (-63%) | 161,603 (-55%) | 25,530 (-21%) | 1,472,875 (-94%) | 213,231 (-72%) | 44,970 (-37%) |
| Infections Prevented/1000 Person-Years |  | 0.29 | 0.05 | -0.004 | 0.73 | 0.12 | 0.02 | 1.08 | 0.16 | 0.03 |
| Life Years Gained/1000* |  | 306 (+0.130%) | 61 (+0.026%) | 11 (+0.005%) | 983 (+0.245%) | 150 (+0.037%) | 29 (+0.007%) | 1,831 (+0.396%) | 287 (+0.062%) | 79 (+0.017%) |
| Total Deaths Averted | B | 156,988 (-26%) | 19,657 (-25%) | 451 (-1%) | 380,171 (-64%) | 37,904 (-48%) | 3,056 (-9%) | 567,911 (-95%) | 53,290 (-67%) | 7,764 (-24%) |
| Deaths Averted/Infection Prevented | B/A | 0.40 | 0.27 | -0.09 | 0.39 | 0.23 | 0.12 | 0.39 | 0.25 | 0.17 |
| DALYs Averted/1000* | C | 532 (-0.224%) | 84 (-0.036%) | -6 (+0.003%) | 1,785 (-0.443%) | 346 (-0.086%) | 114 (-0.028%) | 3,208 (-0.689%) | 590 (-0.127%) | 224 (-0.048%) |
| DALYs Averted/Infection Prevented* | C/A | 1.3 | 1.2 | 1.3 | 1.8 | 2.1 | 4.5 | 2.2 | 2.8 | 5.0 |
| Incremental HIV^†^ Cost/1000 | D | -$7.3 M (-13.6%) | -$1.8 M (-6.7%) | $0.1 M (+0.1%) | -$27.9 M (-40.2%) | -$9.0 M (-20.9%) | -$4.1 M (-4.3%) | -$50.7 M (-67.9%) | -$14.2 M (-29.2%) | -$7.0 M (-7.0%) |
| Incremental ART Cost/1000* | E | $19.7 M (+207%) | -$0.4 M (-6%) | -$0.1 M (-7%) | -$4.0 M (-31%) | -$7.8 M (-70%) | -$2.2 M (-57%) | -$13.2 M (-94%) | -$11.5 M (-93%) | -$4.2 M (-85%) |
| *Value-Based Price/Patient: Curative Therapy* |  | $121,863 | $91,163 | $0** | $439,660 | $134,977 | $53,851 | $1,651,333 | $489,086 | $213,642 |
| Incremental CTx Cost/1000 (using VBP)* | F | $5 M | $5 M | -$0.2 M | $91 M | $28 M | $10 M | $170 M | $45 M | $19 M |
| Incremental HIV+ART+CTx Cost/1000* | D+E+F | $18 M (+27.9%) | $3 M (+8.2%) | $ M (-0.3%) | $59 M (+71.6%) | $11 M (+21.1%) | $4 M (+3.9%) | $106 M (+119.4%) | $19 M (+31.9%) | $7 M (+7.0%) |
| Total Cost per DALY Averted | (D+E+F)/C | $33,016 | $33,016 | $33,016 | $33,016 | $33,016 | $33,016 | $33,016 | $33,016 | $33,016 |

**Table A16. Model Results for the United States**

| Outcomes: CTx+ART vs. ART-Only: United States | Calculation | Pessimistic CTx,  Pessimistic ART | Pessimistic CTx,  Base Case ART | Pessimistic CTx, Optimistic ART | Base Case CTx,  Pessimistic ART | Base Case CTx,  Base Case ART | Base Case CTx, Optimistic ART | Optimistic CTx,  Pessimistic ART | Optimistic CTx,  Base Case ART | Optimistic CTx, Optimistic ART |
| --- | --- | --- | --- | --- | --- | --- | --- | --- | --- | --- |
| Administered CTx Doses, 2030-2040 |  | 258,057 | 617,582 | 590,350 | 550,116 | 778,123 | 828,582 | 913,337 | 855,718 | 803,706 |
| Total Infections Prevented | A | 7,635,726 (-35%) | 2,380,445 (-45%) | -118,395 (+10%) | 15,488,840 (-72%) | 3,927,605 (-74%) | 304,908 (-27%) | 20,798,870 (-96%) | 4,619,402 (-87%) | 512,644 (-45%) |
| Infections Prevented/1000 Person-Years |  | 0.44 | 0.14 | -0.01 | 0.89 | 0.22 | 0.02 | 1.19 | 0.26 | 0.03 |
| Life Years Gained/1000* |  | 8,048 (+0.231%) | 1,998 (+0.057%) | 109 (+0.003%) | 20,568 (+0.422%) | 4,039 (+0.082%) | 326 (+0.007%) | 33,473 (+0.624%) | 7,047 (+0.131%) | 1,391 (+0.026%) |
| Total Deaths Averted | B | 3,603,686 (-35%) | 730,970 (-43%) | 8,224 (-2%) | 7,324,644 (-71%) | 1,137,384 (-66%) | 35,935 (-9%) | 9,978,114 (-97%) | 1,423,882 (-83%) | 112,312 (-29%) |
| Deaths Averted/Infection Prevented | B/A | 0.47 | 0.31 | -0.07 | 0.47 | 0.29 | 0.12 | 0.48 | 0.31 | 0.22 |
| DALYs Averted/1000* | C | 12,177 (-0.347%) | 2,666 (-0.076%) | -415 (+0.012%) | 31,962 (-0.650%) | 7,483 (-0.152%) | 1,346 (-0.027%) | 50,836 (-0.939%) | 11,946 (-0.221%) | 3,096 (-0.057%) |
| DALYs Averted/Infection Prevented* | C/A | 1.6 | 1.1 | 3.5 | 2.1 | 1.9 | 4.4 | 2.4 | 2.6 | 6.0 |
| Incremental HIV^†^ Cost/1000 | D | -$393.1 M (-21.6%) | -$158.1 M (-16.4%) | $9.7 M (+0.4%) | -$1103.8 M (-52.1%) | -$457.0 M (-36.1%) | -$147.3 M (-5.2%) | -$1701.2 M (-76.8%) | -$632.0 M (-46.3%) | -$232.2 M (-7.9%) |
| Incremental ART Cost/1000* | E | $2139.6 M (+428%) | -$60.2 M (-11%) | -$5.4 M (-6%) | -$138.9 M (-21%) | -$568.3 M (-79%) | -$117.3 M (-61%) | -$669.6 M (-95%) | -$740.7 M (-96%) | -$206.5 M (-87%) |
| *Value-Based Price/Patient: Curative Therapy* |  | $0** | $786,019 | $0** | $2,419,892 | $812,280 | $203,667 | $8,702,245 | $3,190,277 | $987,563 |
| Incremental CTx Cost/1000 (using VBP)* | F | -$529 M | $485 M | -$45.9 M | $4,439 M | $1,774 M | $399 M | $7,454 M | $2,567 M | $748 M |
| Incremental HIV+ART+CTx Cost/1000* | D+E+F | $1218 M (+52.6%) | $267 M (+17.4%) | -$42 M (-1.6%) | $3196 M (+115.3%) | $748 M (+37.6%) | $135 M (+4.5%) | $5084 M (+174.3%) | $1195 M (+55.9%) | $310 M (+9.8%) |
| Total Cost per DALY Averted | (D+E+F)/C | $100,000 | $100,000 | $100,000 | $100,000 | $100,000 | $100,000 | $100,000 | $100,000 | $100,000 |

**Table A17. Results of probabilistic sensitivity analysis: Curative Therapy versus ART (base case scenarios for both comparators)**

| Ghana | | | | | | | | | |
| --- | --- | --- | --- | --- | --- | --- | --- | --- | --- |
| PSA Result | **VBP of CTx** | **∆ Total Cost/1000** | **∆ Total Cost %** | **∆ ART Cost/1000** | **∆ ART Cost %** | **∆ DALYs/1000** | **∆ DALYs %** | **∆ Life Years/1000** | **∆ Life Years %** |
| Min | $5,766 | $786,701 | +5.0% | -$18,822,021 | -91% | -59,624 | -7.470% | 670 | +0.085% |
| 2.5% | $7,871 | $1,252,337 | +6.7% | -$5,224,008 | -87% | -17,924 | -2.251% | 1,184 | +0.150% |
| Mean | $12,299 | $3,429,671 | +10.2% | -$2,127,964 | -83% | -6,215 | -0.783% | 3,300 | +0.422% |
| 97.5% | $19,668 | $9,890,468 | +14.8% | -$967,871 | -79% | -2,269 | -0.287% | 9,472 | +1.222% |
| Max | $34,077 | $32,901,307 | +20.3% | -$709,575 | -77% | -1,426 | -0.180% | 37,063 | +5.033% |
| India | | | | | | | | | |
| PSA Result | **VBP of CTx** | **∆ Total Cost/1000** | **∆ Total Cost %** | **∆ ART Cost/1000** | **∆ ART Cost %** | **∆ DALYs/1000** | **∆ DALYs %** | **∆ Life Years/1000** | **∆ Life Years %** |
| Min | $4,350 | $1,996,663 | +1.7% | -$50,613,045 | -85% | -57,360 | -0.232% | 2,966 | +0.012% |
| 2.5% | $5,242 | $2,546,108 | +2.0% | -$17,852,881 | -75% | -12,875 | -0.052% | 3,342 | +0.014% |
| Mean | $6,467 | $3,512,340 | +2.7% | -$12,876,512 | -72% | -8,838 | -0.036% | 4,631 | +0.019% |
| 97.5% | $8,236 | $5,116,341 | +3.8% | -$9,295,015 | -70% | -6,407 | -0.026% | 6,834 | +0.028% |
| Max | $18,860 | $22,795,032 | +8.7% | -$6,042,513 | -69% | -5,024 | -0.020% | 29,202 | +0.118% |
| Kenya | | | | | | | | | |
| PSA Result | **VBP of CTx** | **∆ Total Cost/1000** | **∆ Total Cost %** | **∆ ART Cost/1000** | **∆ ART Cost %** | **∆ DALYs/1000** | **∆ DALYs %** | **∆ Life Years/1000** | **∆ Life Years %** |
| Min | $3,370 | $1,116,090 | +2.8% | -$153,443,462 | -91% | -138,950 | -10.362% | 774 | +0.059% |
| 2.5% | $4,032 | $1,408,885 | +3.3% | -$18,164,291 | -85% | -16,438 | -1.255% | 940 | +0.072% |
| Mean | $5,469 | $2,737,846 | +4.6% | -$8,590,936 | -79% | -5,668 | -0.432% | 2,106 | +0.163% |
| 97.5% | $10,637 | $7,940,190 | +7.3% | -$5,385,934 | -76% | -2,917 | -0.223% | 7,249 | +0.561% |
| Max | $19,873 | $67,119,602 | +10.2% | -$3,943,789 | -76% | -2,311 | -0.177% | 62,660 | +5.331% |
| Nigeria | | | | | | | | | |
| PSA Result | **VBP of CTx** | **∆ Total Cost/1000** | **∆ Total Cost %** | **∆ ART Cost/1000** | **∆ ART Cost %** | **∆ DALYs/1000** | **∆ DALYs %** | **∆ Life Years/1000** | **∆ Life Years %** |
| Min | $3,788 | $2,163,812 | +2.3% | -$309,698,756 | -95% | -787,549 | -10.843% | 4,808 | +0.068% |
| 2.5% | $4,696 | $3,211,807 | +3.3% | -$45,820,398 | -91% | -126,514 | -1.784% | 7,229 | +0.103% |
| Mean | $7,138 | $9,116,218 | +6.1% | -$17,120,026 | -85% | -40,860 | -0.577% | 21,098 | +0.302% |
| 97.5% | $12,047 | $28,226,478 | +11.3% | -$7,519,384 | -81% | -14,396 | -0.204% | 64,070 | +0.920% |
| Max | $22,784 | $175,710,099 | +17.2% | -$4,408,933 | -79% | -9,698 | -0.137% | 428,968 | +6.754% |
|  | | | | | | | | | |
| South Africa | | | | | | | | | |
| PSA Result | **VBP of CTx** | **∆ Total Cost/1000** | **∆ Total Cost %** | **∆ ART Cost/1000** | **∆ ART Cost %** | **∆ DALYs/1000** | **∆ DALYs %** | **∆ Life Years/1000** | **∆ Life Years %** |
| Min | $9,305 | $26,092,151 | +10.1% | -$124,761,661 | -84% | -98,781 | -9.735% | 2,565 | +0.253% |
| 2.5% | $11,177 | $35,457,882 | +12.8% | -$51,670,221 | -81% | -44,069 | -4.275% | 3,549 | +0.351% |
| Mean | $14,347 | $65,619,446 | +17.0% | -$27,632,211 | -77% | -20,326 | -1.980% | 7,318 | +0.735% |
| 97.5% | $19,909 | $142,269,010 | +22.5% | -$16,792,021 | -75% | -10,983 | -1.075% | 17,815 | +1.823% |
| Max | $25,912 | $318,894,661 | +27.9% | -$13,005,897 | -74% | -8,082 | -0.789% | 40,624 | +4.579% |
| Uganda | | | | | | | | | |
| PSA Result | **VBP of CTx** | **∆ Total Cost/1000** | **∆ Total Cost %** | **∆ ART Cost/1000** | **∆ ART Cost %** | **∆ DALYs/1000** | **∆ DALYs %** | **∆ Life Years/1000** | **∆ Life Years %** |
| Min | $2,593 | $436,170 | +1.5% | -$63,758,545 | -93% | -176,433 | -11.171% | 1,642 | +0.109% |
| 2.5% | $3,336 | $731,614 | +2.2% | -$15,294,986 | -91% | -60,359 | -3.926% | 2,466 | +0.164% |
| Mean | $5,350 | $2,223,929 | +3.3% | -$5,165,104 | -86% | -18,550 | -1.217% | 7,864 | +0.529% |
| 97.5% | $8,741 | $7,236,428 | +4.6% | -$2,043,518 | -82% | -6,102 | -0.403% | 26,451 | +1.800% |
| Max | $12,059 | $21,152,600 | +5.9% | -$1,433,866 | -81% | -3,638 | -0.241% | 78,246 | +5.663% |
| Zambia | | | | | | | | | |
| PSA Result | **VBP of CTx** | **∆ Total Cost/1000** | **∆ Total Cost %** | **∆ ART Cost/1000** | **∆ ART Cost %** | **∆ DALYs/1000** | **∆ DALYs %** | **∆ Life Years/1000** | **∆ Life Years %** |
| Min | $4,764 | $1,967,733 | +6.1% | -$96,867,273 | -89% | -68,911 | -11.858% | 1,324 | +0.239% |
| 2.5% | $5,988 | $2,693,734 | +7.2% | -$26,122,515 | -87% | -23,892 | -4.216% | 1,899 | +0.343% |
| Mean | $8,030 | $5,765,156 | +10.0% | -$12,348,592 | -83% | -9,755 | -1.737% | 4,266 | +0.781% |
| 97.5% | $11,843 | $14,119,734 | +13.8% | -$6,774,332 | -80% | -4,558 | -0.817% | 10,639 | +1.973% |
| Max | $15,404 | $40,724,545 | +19.1% | -$4,053,969 | -78% | -3,330 | -0.597% | 30,008 | +5.954% |
| France | | | | | | | | | |
| PSA Result | **VBP of CTx** | **∆ Total Cost/1000** | **∆ Total Cost %** | **∆ ART Cost/1000** | **∆ ART Cost %** | **∆ DALYs/1000** | **∆ DALYs %** | **∆ Life Years/1000** | **∆ Life Years %** |
| Min | $297,352 | $47,914,364 | +19.4% | -$1,143,084,214 | -95% | -36,250 | -3.069% | 519 | +0.044% |
| 2.5% | $352,964 | $64,177,310 | +24.7% | -$201,132,232 | -91% | -7,243 | -0.619% | 685 | +0.059% |
| Mean | $552,471 | $147,089,054 | +36.5% | -$78,366,841 | -85% | -2,673 | -0.229% | 1,501 | +0.129% |
| 97.5% | $981,058 | $398,538,337 | +54.7% | -$34,440,361 | -80% | -1,166 | -0.100% | 3,830 | +0.330% |
| Max | $1,894,224 | $1,994,722,365 | +77.8% | -$26,345,466 | -78% | -871 | -0.075% | 20,087 | +1.756% |
| Germany | | | | | | | | | |
| PSA Result | **VBP of CTx** | **∆ Total Cost/1000** | **∆ Total Cost %** | **∆ ART Cost/1000** | **∆ ART Cost %** | **∆ DALYs/1000** | **∆ DALYs %** | **∆ Life Years/1000** | **∆ Life Years %** |
| Min | $233,867 | $7,083,392 | +20.5% | -$64,767,513 | -85% | -1,157 | -0.188% | 43 | +0.007% |
| 2.5% | $262,849 | $10,455,432 | +25.0% | -$17,970,692 | -72% | -320 | -0.052% | 58 | +0.010% |
| Mean | $314,748 | $16,675,775 | +32.9% | -$10,523,547 | -67% | -189 | -0.031% | 89 | +0.015% |
| 97.5% | $390,629 | $28,146,792 | +43.4% | -$6,393,229 | -64% | -119 | -0.019% | 144 | +0.024% |
| Max | $831,672 | $101,849,634 | +59.0% | -$4,793,097 | -62% | -80 | -0.013% | 576 | +0.094% |
| Italy | | | | | | | | | |
| PSA Result | **VBP of CTx** | **∆ Total Cost/1000** | **∆ Total Cost %** | **∆ ART Cost/1000** | **∆ ART Cost %** | **∆ DALYs/1000** | **∆ DALYs %** | **∆ Life Years/1000** | **∆ Life Years %** |
| Min | $72,996 | $2,542,459 | +6.7% | -$74,029,327 | -84% | -2,175 | -0.485% | 16 | +0.004% |
| 2.5% | $82,795 | $3,330,572 | +8.1% | -$12,354,257 | -72% | -321 | -0.072% | 21 | +0.005% |
| Mean | $104,271 | $6,142,930 | +12.2% | -$6,355,094 | -66% | -123 | -0.027% | 40 | +0.009% |
| 97.5% | $158,315 | $16,056,863 | +21.7% | -$4,110,268 | -64% | -67 | -0.015% | 117 | +0.026% |
| Max | $504,954 | $108,740,744 | +36.3% | -$2,943,055 | -64% | -51 | -0.011% | 949 | +0.213% |
| Spain | | | | | | | | | |
| PSA Result | **VBP of CTx** | **∆ Total Cost/1000** | **∆ Total Cost %** | **∆ ART Cost/1000** | **∆ ART Cost %** | **∆ DALYs/1000** | **∆ DALYs %** | **∆ Life Years/1000** | **∆ Life Years %** |
| Min | $84,872 | $4,196,553 | +11.2% | -$187,293,075 | -84% | -9,651 | -2.373% | 55 | +0.014% |
| 2.5% | $100,196 | $5,978,433 | +14.3% | -$25,363,173 | -78% | -1,388 | -0.344% | 78 | +0.019% |
| Mean | $141,598 | $15,229,763 | +22.1% | -$9,753,098 | -71% | -461 | -0.114% | 200 | +0.050% |
| 97.5% | $223,059 | $45,815,219 | +35.3% | -$4,567,450 | -66% | -181 | -0.045% | 614 | +0.153% |
| Max | $401,325 | $318,653,163 | +48.3% | -$3,388,639 | -64% | -127 | -0.032% | 4,341 | +1.105% |
| USA | | | | | | | | | |
| PSA Result | **VBP of CTx** | **∆ Total Cost/1000** | **∆ Total Cost %** | **∆ ART Cost/1000** | **∆ ART Cost %** | **∆ DALYs/1000** | **∆ DALYs %** | **∆ Life Years/1000** | **∆ Life Years %** |
| Min | $437,468 | $237,330,716 | +18.7% | -$10,677,916,825 | -88% | -154,850 | -3.125% | 1,192 | +0.024% |
| 2.5% | $558,098 | $348,015,893 | +26.2% | -$1,910,496,051 | -86% | -32,851 | -0.668% | 1,884 | +0.038% |
| Mean | $861,549 | $1,002,134,118 | +38.6% | -$710,233,284 | -79% | -10,021 | -0.204% | 5,378 | +0.110% |
| 97.5% | $1,462,819 | $3,285,096,087 | +56.3% | -$307,821,732 | -74% | -3,480 | -0.071% | 17,294 | +0.354% |
| Max | $1,923,782 | $15,484,969,562 | +67.3% | -$209,479,311 | -71% | -2,373 | -0.048% | 78,744 | +1.656% |

*Negative value-based prices, which may occur when DALYs averted are low or positive, are reported as $0.

**Supplement References**

1. Neumann PJ, Sanders GD, Russell LB, Siegel JE, Ganiats TG. Cost-effectiveness in health and medicine: Oxford University Press; 2016.

2. Siebert U, Alagoz O, Bayoumi AM, et al. State-transition modeling: a report of the ISPOR-SMDM Modeling Good Research Practices Task Force-3. *Med Decis Making* 2012; **32**(5): 690-700.

3. World Health Organization. Disability-adjusted life years (DALYs). <https://www.who.int/data/gho/indicator-metadata-registry/imr-details/158> (accessed 11/2/2022.

4. Mohan S, Revill P, Malvolti S, Malhame M, Sculpher M, Kaye PM. Estimating the global demand curve for a leishmaniasis vaccine: A generalisable approach based on global burden of disease estimates. *PLoS Negl Trop Dis* 2022; **16**(6): e0010471.

5. Ochalek J, Lomas J, Claxton K. Estimating health opportunity costs in low-income and middle-income countries: a novel approach and evidence from cross-country data. *BMJ Glob Health* 2018; **3**(6): e000964.

6. Donnell D, Baeten JM, Kiarie J, et al. Heterosexual HIV-1 transmission after initiation of antiretroviral therapy: a prospective cohort analysis. *Lancet (London, England)* 2010; **375**(9731): 2092-8.

7. Hollingsworth TD, Anderson RM, Fraser C. HIV-1 transmission, by stage of infection. *The Journal of infectious diseases* 2008; **198**(5): 687-93.

8. Beacroft L, Hallett TB. The potential impact of a "curative intervention" for HIV: a modelling study. *Glob Health Res Policy* 2019; **4**: 2.

9. Salomon JA, Haagsma JA, Davis A, et al. Disability weights for the Global Burden of Disease 2013 study. *The Lancet Global health* 2015; **3**(11): e712-23.

10. Mikkelsen E, Hontelez JAC, Nonvignon J, et al. The costs of HIV treatment and care in Ghana. *Aids* 2017; **31**(16): 2279-86.

11. Sharma A, Prinja S, Sharma A, Gupta A, Arora SK. Cost of antiretroviral treatment for HIV patients in two centres of North India. *Int J STD AIDS* 2019; **30**(8): 769-78.

12. U.S. Centers for Diseases Control and Kenya Ministry of Health. The Cost of Comprehensive HIV Treatment in Kenya. Report of a Cost Study of HIV Treatment Programs in Kenya. Atlanta, GA (USA) and Nairobi, Kenya, 2013.

13. Bautista-Arredondo S, Colchero MA, Amanze OO, et al. Explaining the heterogeneity in average costs per HIV/AIDS patient in Nigeria: The role of supply-side and service delivery characteristics. *PloS one* 2018; **13**(5): e0194305.

14. Meyer-Rath G, van Rensburg C, Chiu C, Leuner R, Jamieson L, Cohen S. The per-patient costs of HIV services in South Africa: Systematic review and application in the South African HIV Investment Case. *PloS one* 2019; **14**(2): e0210497.

15. Moreland S, Namisango E, Paxton A, Powell R. The costs of HIV treatment, care, and support services in Uganda. *Measure Evaluation, February* 2013.

16. Tucker A, Tembo T, Tampi RP, et al. Redefining and revisiting cost estimates of routine ART care in Zambia: an analysis of ten clinics. *J Int AIDS Soc* 2020; **23**(2): e25431.

17. European Centre for Disease Prevention and Control (ECDC). HIV treatment and care: Monitoring implementation of the Dublin Declaration on Partnership to Fight HIV/AIDS in Europe and Central Asia: 2017 progress report. 2017. <https://www.ecdc.europa.eu/sites/default/files/documents/HIV%20treatment%20and%20care.pdf> (accessed 2/10/2022.

18. McCann NC, Horn TH, Hyle EP, Walensky RP. HIV Antiretroviral Therapy Costs in the United States, 2012-2018. *JAMA Intern Med* 2020; **180**(4): 601-3.

19. Ten Brink DC, Martin-Hughes R, Minnery ME, et al. Cost-effectiveness and impact of pre-exposure prophylaxis to prevent HIV among men who have sex with men in Asia: A modelling study. *PloS one* 2022; **17**(5): e0268240.

20. Wanga V, Peebles K, Obiero A, et al. Cost of pre-exposure prophylaxis delivery in family planning clinics to prevent HIV acquisition among adolescent girls and young women in Kisumu, Kenya. *PloS one* 2021; **16**(4): e0249625.

21. UNAIDS. <https://kpatlas.unaids.org/dashboard>.

22. van Vliet MM, Hendrickson C, Nichols BE, Boucher CA, Peters RP, van de Vijver DA. Epidemiological impact and cost-effectiveness of providing long-acting pre-exposure prophylaxis to injectable contraceptive users for HIV prevention in South Africa: a modelling study. *J Int AIDS Soc* 2019; **22**(12): e25427.

23. Ying R, Sharma M, Heffron R, et al. Cost-effectiveness of pre-exposure prophylaxis targeted to high-risk serodiscordant couples as a bridge to sustained ART use in Kampala, Uganda. *J Int AIDS Soc* 2015; **18**(4 Suppl 3): 20013.

24. Hendrickson C, Long LC, van Rensburg C, et al. The early-stage comprehensive costs of routine PrEP implementation and scale-up in Zambia. *medRxiv* 2021.

25. Durand-Zaleski I, Mutuon P, Charreau I, et al. Costs and benefits of on-demand HIV preexposure prophylaxis in MSM. *Aids* 2018; **32**(1): 95-102.

26. Marcus U, Schmidt D, Schink SB, Koppe U. Analysis of HIV pre-exposure prophylaxis (PrEP) needs and PrEP use in Germany among men who have sex with men. *Z Gesundh Wiss* 2022: 1-17.

27. IrsiCaixa. <https://www.irsicaixa.es/en/news/use-prep-catalonia-could-save-93-million-euros-over-next-40-years>.

28. Kay ES, Pinto RM. Is Insurance a Barrier to HIV Preexposure Prophylaxis? Clarifying the Issue. *Am J Public Health* 2020; **110**(1): 61-4.

29. Rosen J, Asante F. Cost of HIV & AIDS adult and pediatric clinical care and treatment in Ghana: Future Groups, Health Policy Initiative; 2010.

30. Vassall A, Pickles M, Chandrashekar S, et al. Cost-effectiveness of HIV prevention for high-risk groups at scale: an economic evaluation of the Avahan programme in south India. *The Lancet Global health* 2014; **2**(9): e531-e40.

31. Durosinmi-Etti O, Fried B, Dubé K, et al. Sustainability of Funding for HIV Treatment Services: A Cross-Sectional Survey of Patients' Willingness to Pay for Treatment Services in Nigeria. *Glob Health Sci Pract* 2022; **10**(2).

32. Tagar E, Sundaram M, Condliffe K, et al. Multi-country analysis of treatment costs for HIV/AIDS (MATCH): facility-level ART unit cost analysis in Ethiopia, Malawi, Rwanda, South Africa and Zambia. *PloS one* 2014; **9**(11): e108304.

33. Colombie V, Pugliese-Wehrlen S, Deuffic-Burban S, et al. Mean cost of a first combination antiretroviral therapy in HIV-infected patients in France, and determinants of expensive drugs prescription. *Int J STD AIDS* 2012; **23**(12): 865-9.

34. Wolf E, Christensen S, Diaz-Cuervo H. The economic burden of comorbidities among people living with HIV in Germany: a cohort analysis using health insurance claims data. JOURNAL OF THE INTERNATIONAL AIDS SOCIETY; 2018: JOHN WILEY & SONS LTD THE ATRIUM, SOUTHERN GATE, CHICHESTER PO19 8SQ, W …; 2018.

35. Taramasso L, Demma F, Bitonti R, et al. How has the cost of antiretroviral therapy changed over the years? A database analysis in Italy. *BMC health services research* 2018; **18**(1): 691.

36. Lopez-Bastida J, Oliva-Moreno J, Perestelo-Perez L, Serrano-Aguilar P. The economic costs and health-related quality of life of people with HIV/AIDS in the Canary Islands, Spain. *BMC health services research* 2009; **9**: 55.

37. https://www.cdc.gov/hiv/programresources/guidance/costeffectiveness/index.html.

38. Global Burden of Disease Health Financing Collaborator Network. Spending on health and HIV/AIDS: domestic health spending and development assistance in 188 countries, 1995-2015. *Lancet (London, England)* 2018; **391**(10132): 1799-829.

39. US Census Bureau. International Database (IDB). 2022. <https://www.census.gov/data-tools/demo/idb/#/country?COUNTRY_YEAR=2022&COUNTRY_YR_ANIM=2022> (accessed 3/5/2022 2022).

40. World Health Organization. Life tables by country (GHE: Life tables). 2020. <https://www.who.int/data/gho/data/indicators/indicator-details/GHO/gho-ghe-life-tables-by-country> (accessed 5/10/2022 2022).

41. Centers for Disease Control and Prevention. United States Life Tables, 2020. <https://www.cdc.gov/nchs/data/nvsr/nvsr71/nvsr71-02.pdf>.

42. UNAIDS. AIDSinfo. 2020. <https://aidsinfo.unaids.org> (accessed 3/11/2022.
